# Characterising symptomatic substates in individuals on the psychosis continuum: a hidden Markov modelling approach

**DOI:** 10.1017/S003329172500056X

**Published:** 2025-03-12

**Authors:** Isabelle Scott, Emmeke Aarts, Cassandra Wannan, Caroline X. Gao, Scott Clark, Simon Hartmann, Josh Nguyen, Blake Cavve, Jessica A. Hartmann, Dominic Dwyer, Sara van der Tuin, Esdras Raposo de Almeida, Ashleigh Lin, G. Paul Amminger, Andrew Thompson, Stephen J Wood, Alison R. Yung, David van den Berg, Patrick D. McGorry, Johanna T.W. Wigman, Barnaby Nelson

**Affiliations:** 1 Orygen, Parkville, VC, Australia; 2 Centre for Youth Mental Health, The University of Melbourne, Melbourne, VIC, Australia; 3 Department of Methodology and Statistics, Faculty of Social and Behavioural Sciences, Utrecht University, Utrecht, Netherlands; 4 School of Public Health and Preventive Medicine, Monash University, Melbourne, VIC, Australia; 5 Discipline of Psychiatry, Adelaide Medical School, The University of Adelaide, Adelaide, SA, Australia; 6 Telethon Kids Institute, The University of Western Australia, Perth, WA, Australia; 7 Department of Public Mental Health, Central Institute of Mental Health, Medical Faculty Mannheim, University of Heidelberg, Mannheim, Germany; 8 Department of Psychiatry, Interdisciplinary Center Psychopathology and Emotion Regulation, University of Groningen, University Medical Center Groningen, Groningen, The Netherlands; 9 Institute & Department of Psychiatry (LIM-23), Hospital das Clinicas, School of Medicine, University of Sao Paulo, Sao Paulo, Brazil; 10 School of Population and Global Health, The University of Western Australia, Perth, WA, Australia; 11 School of Psychology, The University of Birmingham, Birmingham, UK; 12 Institute for Mental and Physical Health and Clinical Translation, Deakin University, Melbourne, VIC, Australia; 13 Department of Clinical Psychology, Amsterdam Public Health Research Institute, Vrije Universiteit Amsterdam, Amsterdam, The Netherlands; 14 Mark van der Gaag Research Centre, Parnassia Psychiatric Institute, The Hague, The Netherlands

**Keywords:** Hidden markov model, Ambulatory assessment, Ecological momentary assessment, Daily diary study, ultra high risk, clinical high risk, psychosis, psychosis continuum, multilevel modelling, psychosis states, symptom dynamics

## Abstract

**Background:**

To improve early intervention and personalise treatment for individuals early on the psychosis continuum, a greater understanding of symptom dynamics is required. We address this by identifying and evaluating the movement between empirically derived attenuated psychotic symptomatic substates—clusters of symptoms that occur within individuals over time.

**Methods:**

Data came from a 90-day daily diary study evaluating attenuated psychotic and affective symptoms. The sample included 96 individuals aged 18–35 on the psychosis continuum, divided into four subgroups of increasing severity based on their psychometric risk of psychosis, with the fourth meeting ultra-high risk (UHR) criteria. A multilevel hidden Markov modelling (HMM) approach was used to characterise and determine the probability of switching between symptomatic substates. Individual substate trajectories and time spent in each substate were subsequently assessed.

**Results:**

Four substates of increasing psychopathological severity were identified: (1) low-grade affective symptoms with negligible psychotic symptoms; (2) low levels of nonbizarre ideas with moderate affective symptoms; (3) low levels of nonbizarre ideas and unusual thought content, with moderate affective symptoms; and (4) moderate levels of nonbizarre ideas, unusual thought content, and affective symptoms. Perceptual disturbances predominantly occurred within the third and fourth substates. UHR individuals had a reduced probability of switching out of the two most severe substates.

**Conclusions:**

Findings suggest that individuals reporting unusual thought content, rather than nonbizarre ideas in isolation, may exhibit symptom dynamics with greater psychopathological severity. Individuals at a higher risk of psychosis exhibited persistently severe symptom dynamics, indicating a potential reduction in psychological flexibility.

## Introduction

Individuals with attenuated psychotic symptoms are at a heightened risk of developing a full-threshold psychotic disorder. To operationalise the increased risk of transition to psychosis observed in these individuals, the construct of an ultra-high risk (UHR) state for psychosis was introduced (Fusar-Poli et al., 2013; A. R. Yung et al., 1996; Alison R. Yung, Phillips, Yuen, and McGorry, 2004). Despite the heightened risk of transitioning to first-episode psychosis compared to the general population and other clinical populations (de Pablo et al., 2021; Fusar-Poli et al., 2013; Yung et al., 1996; Yung et al., 2003), only approximately 25% of UHR individuals transition over a 3-year period (de Pablo et al., 2021), and over 30% of individuals who present with first-episode psychosis have not experienced a prodromal phase meeting UHR criteria (Shah et al., 2017). In addition, UHR individuals generally exhibit reduced functioning and require treatment irrespective of transition (Mei et al., 2021).

Despite significant heterogeneity in the type, frequency, and trajectories of symptoms that occur in the UHR population (Addington et al., 2011; Lencz, Smith, Auther, Correll, and Cornblatt, 2004; McGlashan, Walsh, and Woods, 2010; Mittal and Addington, 2021; Woods et al., 2023; Yung et al., 2005), they are usually treated with a “one-size-fits-all” approach (Fusar-Poli et al., 2020). Furthermore, individuals lower on the psychosis continuum who exhibit milder symptoms that do not meet UHR criteria may experience distress and impaired functioning (Kelleher et al., 2014; Kelleher et al., 2015; van Os, Linscott, Myin-Germeys, Delespaul, and Krabbendam, 2009). Tools such as the Comprehensive Assessment of At-Risk Mental States (CAARMS; Alison R. Yung et al., 2005) and the Prodromal Questionnaire-16 (PQ-16; Ising et al., 2012) are widely used to assess psychosis risk and provide valuable information about symptom severity and frequency. However, these retrospective tools characterise psychosis symptoms by their average presence or absence, or by the most severe episode, rather than as dynamic and time-varying phenomena and provide little insight into how such experiences may fluctuate in the flow of daily life (Salvi, Rauch, and Baker, 2021). Additional studies are needed to characterise the heterogeneous attenuated psychotic symptoms^1^ and symptom dynamics that occur within individuals on the psychosis continuum to improve treatment and early intervention for these individuals (Mittal and Addington, 2021; Salvi et al., 2021).

To parse the heterogeneity of individuals with attenuated psychotic symptoms, several studies have identified *subgroups* of individuals based on a cross-sectional assessment of symptoms (Allswede et al., 2020; Hartmann et al., 2020; Laloyaux, Larøi, Nuyens, and Billieux, 2018; Valmaggia et al., 2013). However, limited work has explicitly identified clinical *states* or *substates* (considering the presence of attenuated positive symptoms as a state itself) within these individuals. Symptomatic states can be considered as clusters of symptoms that occur within individuals at a similar time (Hamaker, Grasman, and Kamphuis, 2010), for example, the clustering of disinhibition, increased energy, and impulsivity characterising a manic state in bipolar disorder (American Psychiatric Association, 2013).

To investigate the intra-individual temporal association between attenuated psychotic symptoms, autoregressive, linear mixed-effect, and network modelling have been applied to “intensive” longitudinal data from diary studies (van der Steen et al., 2017; van der Tuin et al., 2022). However, these modelling approaches capture pairwise associations, rather than symptomatic states within an individual. Additionally, parameter estimation for these approaches often involves some level of aggregation across time points, losing information regarding intra-individual dynamics over time (Bringmann, Ferrer, Hamaker, Borsboom, and Tuerlinckx, 2018; Hamaker et al., 2010; Scheffer et al., 2024).

Hidden Markov models (HMMs) address these limitations but have not yet been applied to attenuated psychotic symptoms. The HMM is a latent longitudinal mixture modelling approach, which uncovers empirically derived symptom substates from longitudinal data and determines the probabilities of switching between substates over time (Hamaker et al., 2010). A previous study applied an HMM to diary data and uncovered empirically derived neutral, elevated, mixed, and lowered states in patients with bipolar disorder (Mildiner Moraga et al., 2024). By pairing the HMM with a multilevel framework, we can infer symptom patterns shared among individuals, and evaluate individual-specific symptom dynamics (Hale and Aarts, 2023). This may offer new insights into the heterogeneity of symptom dynamics that occur within individuals on the psychosis continuum.

As is the case with many elements of psychopathology, true discrete symptomatic states may not exist (Haslam, Holland, and Kuppens, 2012). Nonetheless, assuming some general patterns exist, discretising the problem can facilitate interpretation and aid clinical decision-making (Eaton et al., 2023; McGorry and Hickie, 2019). The current study applied a multilevel HMM to daily diary data to identify and characterise symptomatic substates in individuals on the psychosis continuum. Furthermore, the time spent within and the pattern of how individuals transition between substates was examined, including an exploration of whether this varies by point of severity along the psychosis continuum. We hypothesised that individuals further along the psychosis continuum would have a tendency to spend time in substates with multiple and more severe co-occurring symptoms.

## Materials and methods

### Sample

The present study utilises data collected at baseline from the Mirorr study, a Dutch daily diary study conducted with 96 participants aged 18–35 years, who exhibited varying levels of attenuated psychotic symptoms. Participants completed a 90-day daily diary study at baseline and at a 1-year follow-up. Participants are also scheduled to receive yearly follow-up questionnaires regarding functioning and clinical stage for 3 years. A detailed description of the study is provided in the protocol paper (Booij et al., 2018). Individuals were split into four subgroups, increasing in clinical severity:

1. *Non-Clinical* (n = 25)*:* Individuals recruited from the general population and not receiving mental health care at baseline. From the 100 individuals recruited, the 25% with the highest scores on the positive symptoms subscale of the Community Assessment of Psychic Experiences (CAPE) were selected (Konings, Bak, Hanssen, van Os, and Krabbendam, 2006; Versmissen et al., 2008).

Subgroups 2–4 were all receiving mental health care at baseline.

1. *Mild-PLE (psychotic-like experience)* (n = 27): Individuals who had a total score of less than six on the Prodromal Questionnaire-16 (PQ-16), a screening tool for identifying individuals at clinical high risk for psychosis (Ising et al., 2012).
2. *Mod-PLE* (n = 24): Individuals who scored six or above on the PQ-16 but did not meet UHR criteria as defined by the Comprehensive Assessment of At Risk Mental States (CAARMS; Alison R. Yung et al., 2005). A cut-off of six on the PQ-16 has previously been found to achieve the best balance between sensitivity and specificity in classifying UHR cases (Ising et al., 2012).
3. *UHR* (n = 20): Individuals who met UHR criteria as defined by the CAARMS, excluding trait-only criteria.

In addition, the following measures were used to measure psychopathology and functioning across the entire sample:

*Subclinical psychotic experiences* were measured with the Community Assessment of Psychic Experiences (CAPE; Konings et al., 2006), a self-report questionnaire consisting of 42 items. A 4-point Likert scale is used to score both the frequency and distress of psychotic experiences. The CAPE has good reliability and validity (Konings et al., 2006) and was found to have good internal consistency in the present sample (Cronbach’s α = .89) (van der Tuin et al., 2022).

*General psychopathology* was measured using the Dutch Symptom Checklist Revised (SCL-90-R), a 90-item self-report questionnaire measuring psychological symptoms in the past week. The Dutch SCL-90 has high reliability (ω = .98) (Smits, Timmerman, Barelds, and Meijer, 2015) and was found to have excellent internal consistency in the present sample (Cronbach’s α = .98) (van der Tuin et al., 2022).

*Social functioning* was measured with the Groningse Vragenlijst voor Sociaal Gedrag (GVSG) (De Jong and Lubbe, 2001), a Dutch self-report questionnaire. The GVSG includes nine social domains: five domains assessing interpersonal relationships and four domains assessing social and role functioning. Each domain includes five questions, scored on a 4-point scale, and only domains relevant to the individual are included in the total score calculation.

The study was approved by the medical ethical committee of the University Medical Centre Groningen, Groningen, The Netherlands (registration number MEC no. 2015/159, ABR no. NL52974.042.15). All participants provided written informed consent.

### Daily diary data

Participants completed an 81-item questionnaire (Supplementary Table S1) on their smartphone each evening for 90 days. Six items were included in the HMM, selected after study completion to capture attenuated psychotic (four items) and affective symptoms (two items). Translated from Dutch to English, these included “I felt suspicious today” (suspiciousness), “I felt that others could read my thoughts today” (broadcasting), “Today I had the feeling that others did not like me” (disliked), “I felt that others could control me today” (external control), “How stressed were you today?” (anxiety) and “I felt down today” (mood). All six items were rated on a scale of 0–100, depending on the degree to which the participant agreed with the item statement (not at all - very much).

A further two diary items, rated on a scale of 0–7, recorded perceptual disturbances: “Today I heard voices that others couldn’t hear” (auditory hallucinations) and “Today I saw things that others couldn’t see” (visual hallucinations). The low endorsement of these two hallucination-related items in this population—reflecting earlier stages on the psychosis continuum—resulted in insufficient variability in the data to reliably estimate corresponding HMM parameters. As such, these two items were not included in fitting the HMM but were included in post-hoc analyses.

While a further six Mirorr items (30, 37–40, and 46; Supplementary Table S1) were potentially related to psychotic experiences (delusions or disorganised thoughts), these items were excluded due to possible alternative interpretations (e.g. “Today I felt special”). Exploratory data analysis further supported this decision, as these items were often endorsed in the absence of any other psychotic experiences. The final six items related to psychotic experiences included in the present analyses (four within the HMM and two within post-hoc analyses) are comparable to those commonly used to measure psychotic experiences in previous studies (Myin-Germeys, Marcelis, Krabbendam, Delespaul, and van Os, 2005; Oorschot, Kwapil, Delespaul, and Myin-Germeys, 2009; Raposo De Almeida et al., 2024). Only two items related to affect were included in addition to these six items related to psychotic experiences, aligned with the primary study objective to determine empirically derived symptomatic substates in individuals along the psychosis continuum. These included one item related to mood and one related to anxiety/stress, symptoms that commonly co-occur with psychotic experiences (Hartley, Barrowclough, and Haddock, 2013). By carefully selecting a small pool of theoretically motivated and pragmatically guided items, the current study prioritised substate interpretability and minimised the risk of overfitting. The remaining 67 items excluded from the present analyses measured experiences nonspecific to psychosis (e.g. affect, sleep or social events).

### Statistical analysis

A Bayesian multilevel HMM (Altman, 2007; Kirchherr et al., 2023; Zhang and Berhane, 2014) was fitted to the diary data using the statistical software R (Core Team, 2021) version 4.2.3 and the *mHMMbayes* (Aarts, n.d.) package version 1.0.0.

#### Multilevel HMM

A description of HMMs and related modelling assumptions are provided in the Supplementary Material (Supplementary Methods; Supplementary Figure S1). Two sets of parameters describe the HMM: (1) The emission distribution parameters define a multivariate Gaussian density function indicating the probability of observing certain diary item scores given the current substate, (2) The transition probabilities for switching from one substate to another at the subsequent time point.

The multilevel HMM is a hierarchical model which includes random effects for the emission distribution parameters and transition probabilities to capture differences in participants’ symptom dynamics. Subsequently, the meaning of each substate can be preserved (the ordinality of values across the substates is consistent across individuals) while accommodating individual differences. For example, while the item “I felt down” may be rated most highly by individuals when they are in a depressed substate, the mean item score for this substate is likely to vary across individuals. Furthermore, participant-level parameters are regularised, pooling them toward the group-level means and making the model more robust to outliers (Gelman et al., 2013).

#### Missing data

There was an average of 6.9 (maximum four consecutive) missing days across participants. Missing data was assumed missing at random (Little and Rubin, 2019).

#### Parameter estimation

The substate transition probabilities and emission parameters were estimated using a Bayesian procedure, employing a hybrid Metropolis within Gibbs MCMC algorithm (Aarts, 2019; Scott, 2002). The *depmixS4* (Visser and Speekenbrink, 2010) package was first used to train a single-level HMM, providing initial parameter values for the Bayesian estimation procedure. Weakly informative hyperpriors were specified for the emission distributions to allow the data to primarily inform the parameter estimates (see Supplementary Methods hyperprior values). The model was fitted using 4000 iterations of the MCMC sampler with the first 1000 iterations treated as a burn-in period. Bayesian multiple imputation is able to handle occasional missing data (missing at random) without introducing bias (de Haan-Rietdijk et al., 2017). For missing observations, at each iteration, the forward probability of the corresponding hidden states is computed and a new set of hidden states is sampled from the posterior distribution. Convergence of all sample-level parameters was checked with the multivariate potential scale reduction factor, utilising a threshold of 1.05 (Rhat ≤1.05), and calculated using two additional chains with randomised starting values (Brooks and Gelman, 1998).

#### Model fit

Separate multilevel HMMs were trained for 2–6 states, including and omitting the affective symptom items. The latter was done to ascertain whether the substates remained stable when excluding affective symptoms. This ensures that the substate determination is driven by psychotic symptoms, while allowing for the assessment of how affective symptoms vary across these psychotic substates.

Model selection was based on Akaike information criterion (AIC) (Akaike, 1974), the ability to reproduce the original empirical data through post-hoc model-based simulations (posterior-predictive checks) (Gelman et al., 2013), and the evaluation of pseudo-residuals. Posterior predictive checks for the group-level emission distributions were performed by generating 500 simulated 90-day time series datasets for 96 hypothetical individuals. Posterior predictive checks for the individual-level emission distribution and transition parameters were performed by generating 500 simulated 90-day time series datasets for 96 hypothetical individuals. The individual-level emission distribution parameters and transition probabilities were provided as input. Residuals for each individual were calculated separately for each diary item by subtracting the individual-level item mean score from the 90-day daily item scores. Residuals were assessed for homoscedasticity and a zero mean to ensure the model fit was unbiased across time. Residuals were also aggregated across diary items and time for each individual, and further assessed for zero mean centring.

#### Determining individual substate sequences

Using fitted participant-level parameters (posterior means of the individual level parameters; see *Parameter Estimation* above) and the Viterbi algorithm, the most likely state sequence for each participant over the 90-day assessment period was estimated based on their observed data (Forney, 1973; Viterbi, 1967).

#### Post-Hoc analysis of perceptual disturbances

Scores for the two perceptual disturbance items were transformed to indicator variables, taking a daily value of zero (score < 4) or one (score ≥ 4).

#### Difference in substate switching patterns between subgroups

The multilevel HMM employs a multinomial logit framework to model the transition probabilities between substates, allowing for the inclusion of random effects and optional covariates. To examine how transitions between substates vary with psychosis risk, we adapted the model to include subgroup as a participant-level logit regression covariate influencing transition probabilities. This allowed us to investigate how subgroup membership impacted the likelihood of switching between substates while maintaining consistent substate characterisations (emission distributions) as in the model without covariates.

Since the mHMMbayes package does not natively handle categorical variables, we represented subgroup membership using multiple binary (0/1) variables. For interpretability, logits for the subgroup-level transition probabilities were transformed back into probability space for each MCMC iteration. From these iterations, excluding the burn-in period, we calculated the posterior mean and associated 95% credible interval for all subgroup transition probabilities.

## Results

The mean (SD) age of individuals in the study was 24.7 (4.2) years and 76% of individuals were female. Psychological distress, psychotic experiences, and impairments in social functioning increased across the subgroups. There was no significant difference in the number of days of missing diary data between subgroups (Table 1).

**Table 1.** Demographic and clinical characteristics of the sample

	Subgroup					
	Non-Clinical (N = 25)	Mild-PLE (N = 27)	Moderate-PLE (N = 24)	UHR (N = 20)	Total sample (N = 96)	Significant difference
**Demographic**						
Age, mean (SD)	23.3 (3.4)	24.8 (4.0)	26.1 (4.1)	24.8 (5.3)	24.7 (4.2)	—
Gender (% female)	80	74.1	70.8	80	76	—
Completed Education						
Low (%)	4	18	8	30	14	—
Middle (%)	56	52	58	50	54	—
High (%)	40	26	29	20	29	—
Other (%)	0	4	4	0	2	NA
Recruitment Location in Netherlands (%)						
North	100	100	25	40	79	1,2 > 4 > 3
Central/South	0	0	75	60	21	NA
**Clinical Functioning**						
SCL–90-R, mean (SD)	141.4 (38.2)	173.8 (45.1)	211.0 (56.1)	232.5 (57.3)	186.9 (59.3)	4,3 > 2, 1
GVSG, mean (SD)	15.5 (1.4)	14.6 (1.6)	13.3 (2.0)	13.5 (1.7)	14.3 (1.9)	1 > 3, 4;
CAPE, mean (SD)						
Total	60.9 (9.6)	66.5 (11.7)	77.4 (15.4)	75.2 (17.3)	69.6 (14.9)	4,3 > 1; 3 > 2
Positive symptoms	23.5 (3.8)	22.4 (2.2)	26.2 (5.1)	26.6 (5.3)	24.5 (4.4)	4,3 > 2; 4 > 1
Negative symptoms	23.8 (5.1)	27.2 (7.4)	32.5 (7.8)	30.9 (9.5)	28.4 (8.1)	4,3 > 1
Depressive symptoms	13.6 (3.3)	16.9 (4.8)	18.6 (5.5)	17.7 (5.4)	16.6 (5.1)	4,3 > 1
Missing Diary Days, mean (SD)	6.1 (5.2)	7.2 (6.0)	6.5 (5.0)	8.0 (5.2)	6.9 (5.3)	—

*Significant difference: *P* < .05. Group differences for continuous variables were determined using analysis of variance with Tukey test for pairwise comparisons. Group differences for proportions were determined using chi-square tests with Holm adjustment. SCL-90-R, Dutch Symptom Checklist Revised; CVSG, Groningse Vragenlijst voor Sociaal Gedrag; CAPE, Community Assessment of Psychic Experiences.

### Number of substates

Balancing model fit, parsimony, and interpretability, the multilevel HMM consisting of four substates was selected as the most appropriate representation of the data (Pohle, Langrock, van Beest, and Schmidt, 2017). The AIC favoured the four-substate model over the two- and three-substate models (Supplementary Figure S2) and the four-substate model was able to adequately describe the empirical data at both the group and individual level (Supplementary Figures S3-S7). While increasing the number of groups beyond four resulted in a slightly lower AIC, the reduction was marginal and did not justify the added complexity. The structural differences between the five-state and four-state models were minimal and did not provide meaningful additional insights: four states in the five-state model were nearly identical to those of the four-state model, with the additional fifth state largely overlapping with the most clinically severe state in the four-state model, differing only by a slight leftward shift in the emission distribution. For a detailed description of the emission distribution in the four-substate model, see the Substate Compositions section below; a comparison with the five-state model is provided in Supplementary Figure S8.

### Substate compositions

**Figure 1.**
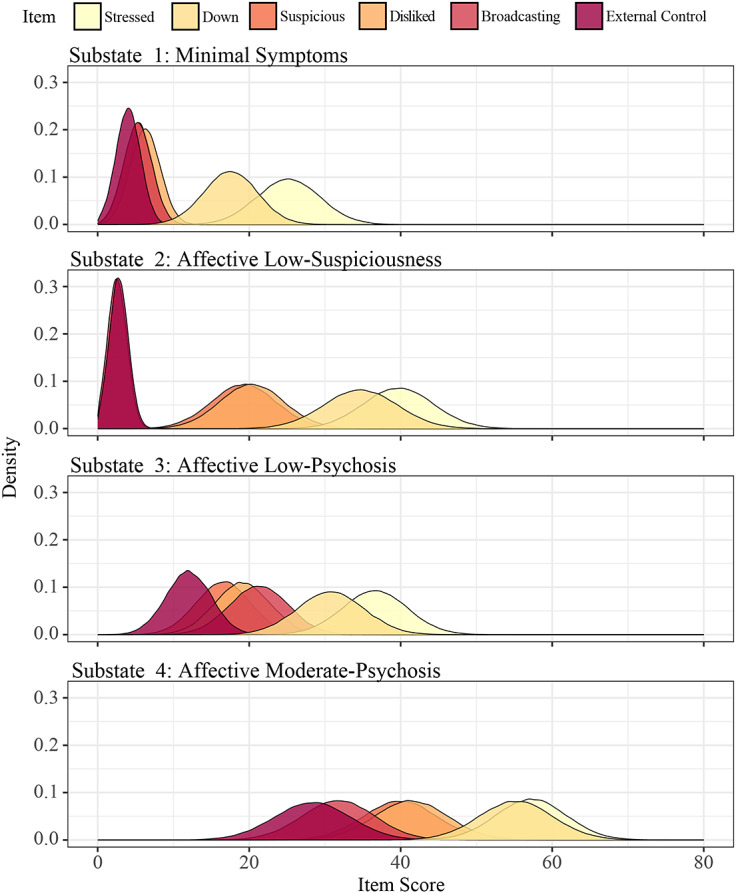
Group level emission distributions characterizing the four clinical substates uncovered with the multilevel HMM. A continuous multivariate normal distribution captures the most likely scores for each of the six diary items that are observed in each substate.

The symptomatic profiles of the four substates are captured by the four group-level emission distributions visualised in Figure 1. These probability distributions describe how likely (vertical axis) individuals are to report specific item scores (horizontal axis) within each substate, with higher scores along the horizontal axis corresponding to more severe symptoms. As such, the group-level emission distributions provide insight into the severity and co-occurrence of symptoms across states. The first substate (Minimal Symptoms) is characterised by negligible (mean < 10) scores across the four psychotic items, and low scores (10 < mean < 25) for the affective items, suggesting mild mood disturbances. The second substate (Affective Low-Suspiciousness) is characterised by low scores for suspiciousness and feelings of being disliked, coupled with moderate scores for affective symptoms (25 ≤ mean < 60). This substate suggests a decoupling of psychotic symptoms, with suspiciousness co-occurring alongside feelings of being disliked but in the absence of similarly severe thoughts of broadcasting or external control. The third substate (Affective Low-Psychosis) is characterised by low scores across all psychotic items, with the highest scores for broadcasting, and moderate affective symptoms. As such, the third substate is similar to the second, differentiated predominantly by the additional presence of thoughts of external control and broadcasting. The fourth substate (Affective Moderate-Psychosis) represents the most clinically severe substate, exhibiting moderate (25 < mean < 75) scores across all items (affective and psychotic). There was heterogeneity in the individual emission distribution means, however, the substate characterisations were consistent with the group-level emission findings (variable-dependent ordinal relation over states was preserved across individuals; Supplementary Figure S9).

Analysis of the emission distributions across all four substates highlights consistent patterns of symptom co-occurrence, with suspiciousness and feelings of being disliked, as well as broadcasting and external control, emerging as commonly co-occurring symptom pairs. Additionally, suspiciousness and feelings of being disliked occurred in isolation within the second substate, without accompanying unusual thought content or passivity phenomena. Conversely, thoughts of broadcasting and external control only tended to occur in the context of suspiciousness and feelings of being disliked, suggesting an asymmetric relationship between these symptom clusters.

### Substate trajectories

**Figure 2.**
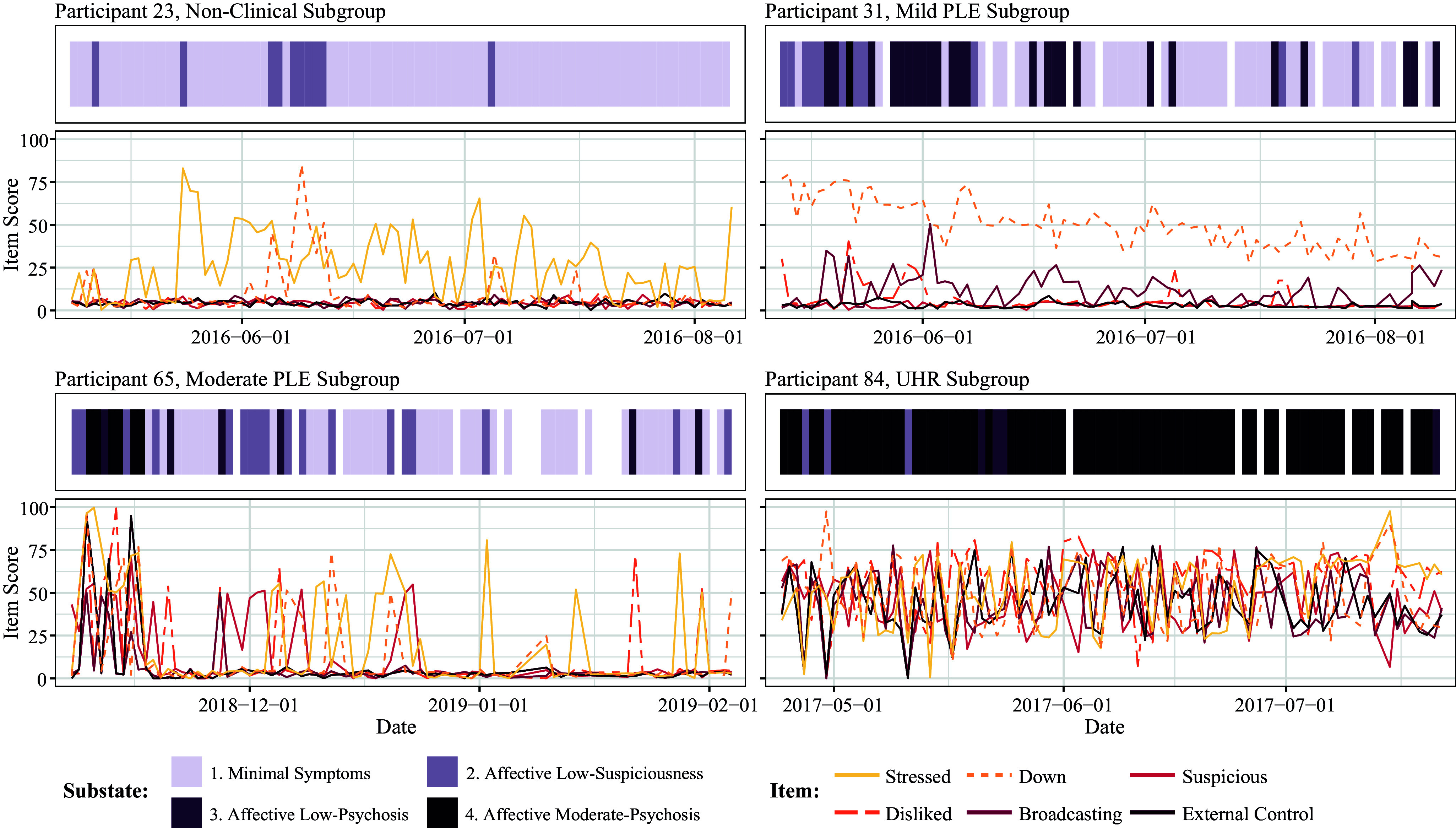
Substate sequences generated for four individuals across subgroups 1–4. Missing diary data is imputed while missing substate sequence data is displayed in white. The exemplar individual spent: (a) most time in the first substate, consistent with negligible psychotic symptoms; (b) most time in the first and third substates, displaying high affective symptoms throughout the assessment period, with occasional symptoms of broadcasting; (c) multiple days in the fourth substate at the start of the assessment period, displaying high psychotic symptoms during this time. In the middle of the assessment period, this same individual spent periods in the second substate, displaying periods of high affective symptoms and suspiciousness, and, towards the end of the assessment period, spent most time in the first substate, exhibiting minimal psychotic symptoms; (d) most time in the fourth substate, displaying moderate scores for all items across the assessment period.

The diary data and substate sequence for four exemplar individuals, chosen due to their unique substate patterns, are displayed in Figure 2. The substate sequences for the full set of individuals are provided in the Supplementary Material (Supplementary Figures S10 and S11). Individuals displayed a large degree of intra-individual heterogeneity; on average, individuals switched substates 37 times (SD = 14) over the assessment period. There was no significant difference in the state-switching frequency between subgroups (ANOVA, *P* = .19).

### Relationship between individual states and perceptual disturbances

**Figure 3.**
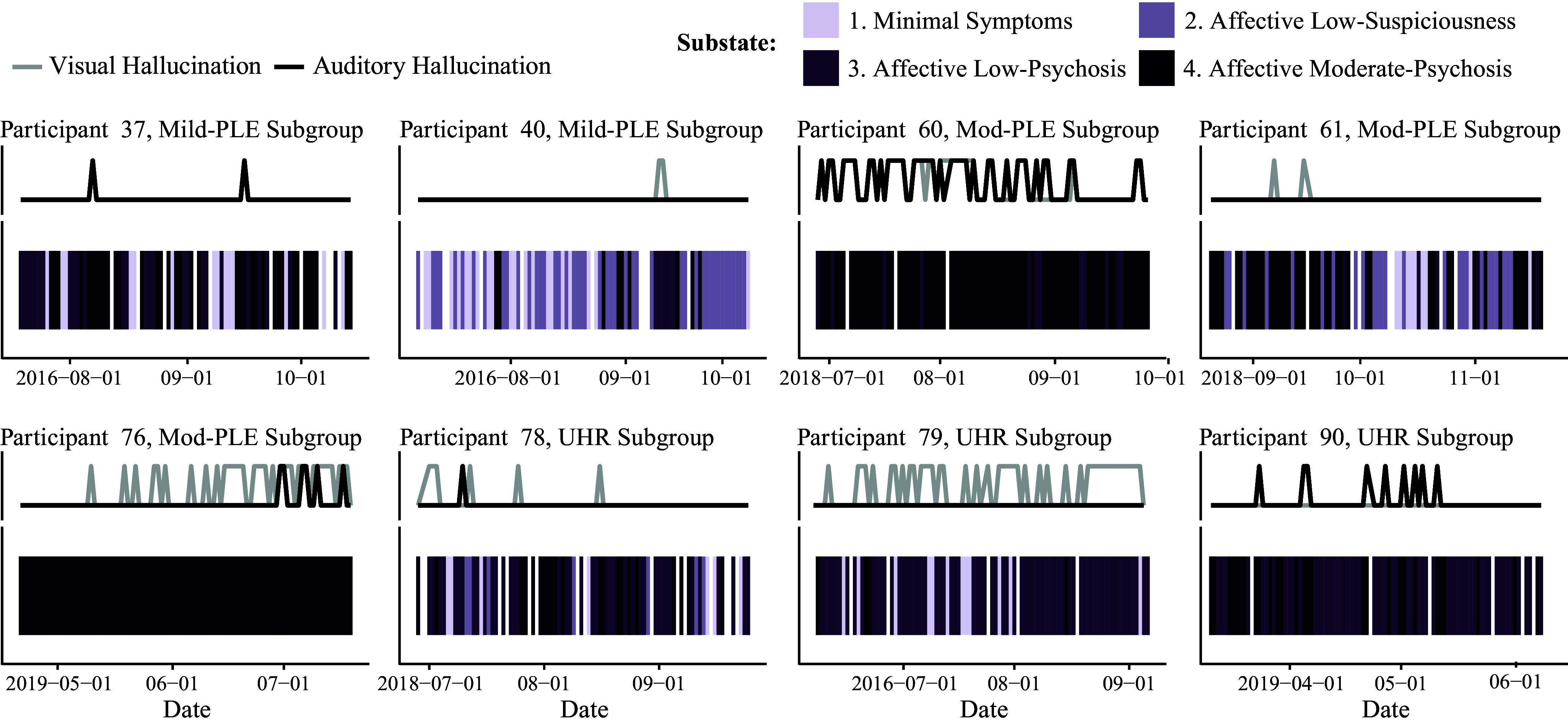
Perceptual disturbances and temporally associated state sequences for eight individuals belonging to subgroups 2–4. Grey and black lines (top panel) depict whether a perceptual disturbance was present or absent for each day across the 90-day assessment period. Missing state sequence data is displayed in white.

Of the 148 occurrences of perceptual disturbances across individuals, 140 occurred when an individual was in the third or fourth substate (s1: Minimal Symptoms = 7, s2: Affective Low-Suspiciousness = 1, s3: Affective Low-Psychosis = 47, s4: Affective Moderate-Psychosis = 93), indicating broadcasting and concerns regarding external control tend to accompany perceptual disturbances. The state sequences for the individuals who recorded a perceptual disturbance on two or more are displayed in Figure 3. The state sequence is coupled with a time series indicating perceptual disturbance occurrences.

### Substate switching patterns

**Figure 4.**
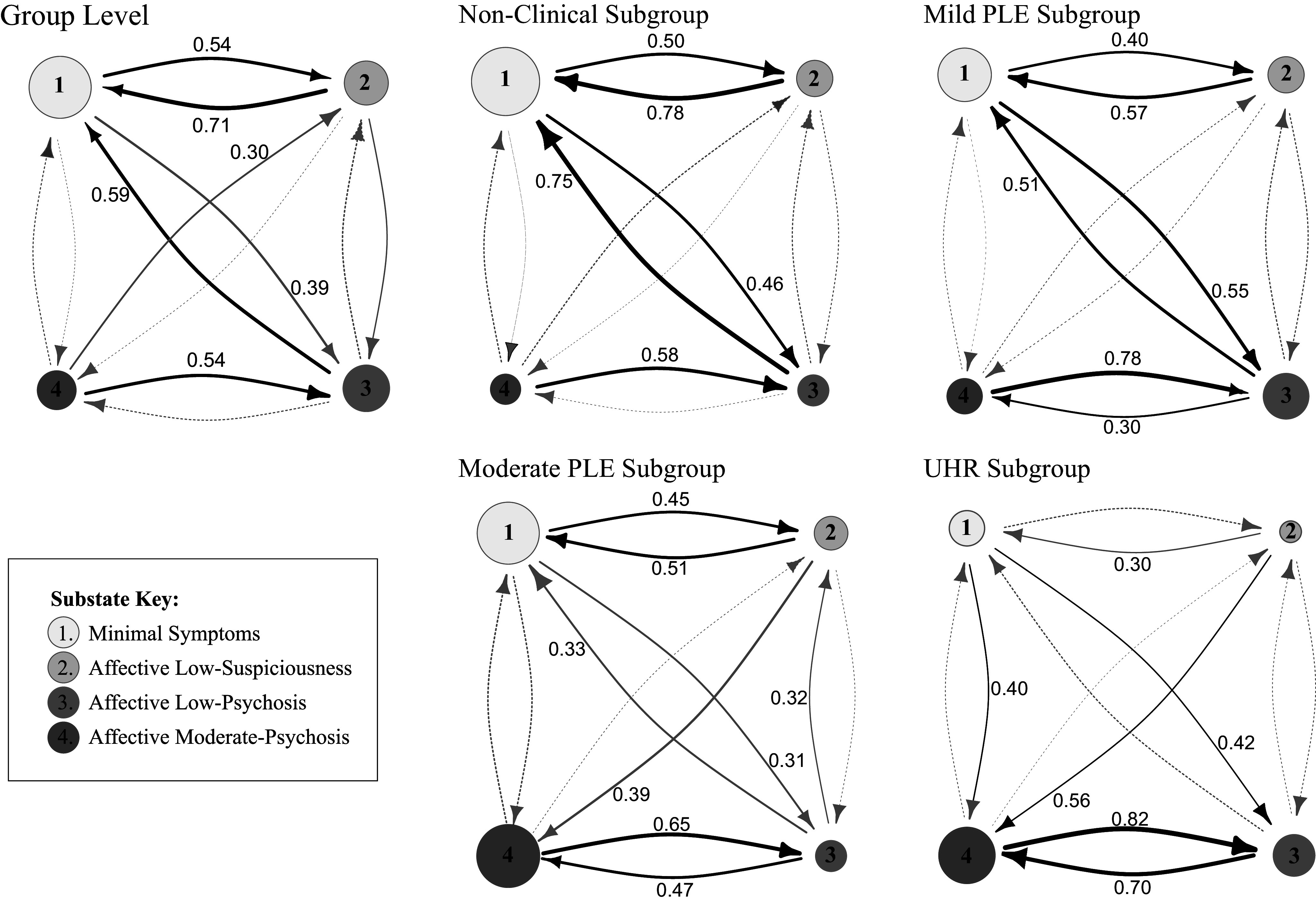
Network diagram of group-level and subgroup substate switching patterns. Switching probabilities are calculated as the posterior mean of estimates generated by the MCMC sampler, excluding the burn-in period. Nodes represent substates, and edges represent relative switching probabilities. The node area is proportional to the probability of an individual remaining in that substate. Edge thickness is proportional to the size of the relative switching probability (dashed: probabilities <.3, grey: probabilities 2–.4, black: probabilities >.4).

The group- and subgroup-level state switching patterns are displayed in Figure 4. Bayesian credible intervals for the transition probabilities for each subgroup are provided in the Supplementary Material (Supplementary Table S2), as well as pairwise differences in stability and switching probabilities, based on nonoverlapping credible intervals (Supplementary Table S3). At the group level, the first Minimal Symptoms substate is the most stable, as indicated by a larger node compared to other substates. This suggests that when an individual is currently within this substate, they are likely to remain there at the next point in time. However, at the subgroup level, the first Minimal Symptoms substate exhibits lower stability (indicated by a smaller first node) for the UHR subgroup than the other three subgroups, particularly when contrasted with the first nonclinical subgroup. This suggests that individuals within the UHR subgroup are more likely to transition out of the first Minimal Symptoms substate at the next time point, resulting in less sustained periods of minimal symptoms.

The probability of switching from the third back to the first substate also varies across subgroups. Individuals in the first nonclinical and second mild-PLE subgroups display a higher probability of returning to the first Minimal Symptoms substate when in the third Affective Low-Psychosis substate, as indicated by thick black arrow linking the third with the first substate. This suggests that individuals within the first nonclinical and second mild-PLE subgroups are *less* likely to exhibit sustained periods of multiple low-to-moderate psychotic symptoms. In contrast, individuals in the third and fourth subgroups (moderate-PLE and UHR) are *more* likely to progress from the Affective Low-Psychosis substate to the more severe Affective Moderate-Psychosis substate, than to return to the Minimal Symptoms or Affective Low-Suspiciousness substates. UHR individuals exhibit a strong bidirectional relationship between the third (Affective Low-Psychosis) and fourth (Affective Moderate-Psychosis) substates, as indicated by the thick black arrows linking these substates. UHR individuals also exhibit a high probability of switching into these substates from the first (Minimal Symptoms) and second (Affective Low-Suspiciousness) less severe substates. Consequently, UHR individuals may get “trapped” moving back and forth between the two most severe substates, as evidenced by their individual substate sequences (Supplementary Figures S10 and S11) and the time UHR individuals spent in these two substates across the assessment period (Supplementary Figure S12).

## Discussion

To our knowledge, this is the first study to identify symptomatic substates in individuals early on the psychosis continuum, providing novel insights into how symptoms interact and evolve over time. It is also, to our knowledge, the first study to characterise and investigate the temporal symptom dynamics of psychotic symptoms (attenuated or frank) by utilising a multilevel HMM approach. Symptomatic substates were inferred from the response patterns of 96 individuals over four daily diary items related to attenuated psychotic symptoms and two related to affective symptoms. This modelling approach provided evidence for four symptomatic substates: increasing in severity: (1: Minimal Symptoms), characterised by low-grade affective and negligible psychotic symptoms; (2: Affective Low-Suspiciousness), marked by low levels of suspiciousness and feelings of being disliked, with moderate affective symptoms; (3: Affective Low-Psychosis), involving low levels of suspiciousness, feelings of being disliked, and thoughts of broadcasting and external control, with moderate affective symptoms; and (4: Affective Moderate-Psychosis), characterised by moderate levels of suspiciousness, feelings of being disliked, thoughts of broadcasting and external control, and affective symptoms. Individuals switched frequently between substates, highlighting the dynamic nature of psychopathology.

Temporal dynamics discerned from the multilevel HMM results were compared across the four attenuated psychotic symptom subgroups to link these dynamic findings to more traditional cross-sectional severity measures and provide a complementary dynamic perspective. Clear trends emerged across the subgroups in terms of their substate dynamics. Consistent with our original hypothesis, with increasing psychosis risk, individuals were more likely to transition into and stay in substates characterised by more severe and multiple psychotic symptoms, with difficulty switching back into or remaining in substates with fewer or negligible psychotic symptoms. While this dynamic finding was hypothesised, it could not be assumed, as the CAARMS definition of UHR does not require the presence of multiple co-occurring symptoms. Instead, the CAARMS treats symptoms as disjunctive entities, meaning that individuals can be classified as at UHR for psychosis if they meet severity and frequency criteria for any single symptom. In contrast, the present findings indicate that individuals at UHR of psychosis frequently occupy substates characterised by *multiple co-occurring* symptoms (e.g. suspiciousness, paranoia/feelings of being disliked, thoughts of broadcasting and external control), highlighting the potential importance of considering symptom co-occurrence in understanding psychosis risk dynamics.

The persistence of psychotic symptoms in populations at greater risk, especially passivity experiences, may reflect an increase in psychological inflexibility (Morris and Mansell, 2018). It is also consistent with lived experience accounts that detail a reduction in the individual’s ability to shift away from these experiences as psychosis progresses (Fusar-Poli et al., 2022). Clinically, this suggests that there may be a greater tendency to return (“relapse”) into these more severe symptomatic substates in response to situational/contextual stressors, so strategies to develop resilience, stress management, and ease to re-entry into clinical services may be warranted. Diary studies could serve as a valuable monitoring or psychoeducation tool, helping individuals track changes in and understand their dynamic response to stressors.

Despite differences in the clinical population, analytical approach, and the symptom severity recorded (attenuated rather than frank psychotic symptoms), the findings of this study echo and extend previous cross-sectional work investigating the co-occurrence of symptoms in first-episode psychosis (Lemonde et al., 2021). Along with suspiciousness feelings of being disliked – which, at the group level, potentially represent a level of paranoia as indicated by this item’s close tracking with suspiciousness – was the most common psychotic symptom observed in the present study, occurring at low and moderate levels in three of the four substates. Broadcasting and delusions of external control, but not suspiciousness or feelings of being disliked/paranoia, were positively correlated with perceptual disturbances. Further, similar levels of stress characterised the second and third substates, despite the presence of broadcasting only in the latter substate, supporting the finding of Lemonde (2021) that broadcasting and anxiety are not specifically correlated.

The current study identified suspiciousness and feelings of being disliked/paranoia, and broadcasting and external control, as commonly co-occurring symptom pairs. This co-occurrence aligns with the clinical grouping of these phenomena into paranoid delusions and passivity phenomena, and the categorisation of these into nonbizarre ideas/suspiciousness and unusual thought content within the CAARMS and PSYCHS. However, in contrast to these measures, which apply equal weighting to nonbizarre ideas and unusual thought content when defining UHR and transition criteria, the present study identified an asymmetric association between these symptom classes. Broadcasting and delusions of external control only tended to occur in the context of symptoms of suspiciousness and paranoia/feelings of being disliked (within the third, Affective Low-Psychosis, and fourth, Affective Moderate-Psychosis, substates). In contrast, suspiciousness and paranoia/feelings of being disliked also commonly occurred in isolation (within the second Affective Low-Suspiciousness substate). This asymmetric association is consistent with previous work (Paquin et al., 2023) and suggests that individuals who report unusual thought content may exhibit more severe psychopathological dynamics (in terms of the number and severity of co-occurring symptoms). The present finding that unusual thought is correlated with more severe psychopathological dynamics also supports previous work which identified baseline levels of unusual thought content to be a stronger predictor of psychosis onset in UHR individuals than suspiciousness or paranoia (Mason et al., 2004; Thompson, Nelson, and Yung, 2011). While further work is required to confirm the present findings, taken together with previous work, these findings have important implications for staging. Specifically, in line with the contention put forward by Mason et al. (2004) that at-risk criteria are too disjunctive, these findings suggest that assessment tools could be further refined to consider the number and type of co-occurring symptoms and to consider symptom-specific severity thresholds.

The asymmetric association between psychotic symptoms observed in this study has important implications for the refinement and development of future risk calculators. The asymmetric association indicates a possibly complex and nonlinear correlation structure between attenuated psychotic symptoms, suggesting care should be taken regarding feature selection for risk calculators. Such nonlinear correlations can be missed with traditional methods for detecting and addressing multicollinearity (Armstrong, 2019; Bollinger, Belsley, Kuh, and Welsch, 1981). Furthermore, this finding supports the use of nonlinear features or models, such as decision trees, even in the setting of few predictor variables (Pudjihartono, Fadason, Kempa-Liehr, and O’Sullivan, 2022).

The present study should be viewed in the context of several limitations. The study utilised daily diary entries, which offer several advantages, including the ability to capture real-time data and reduce recall bias, but may introduce biases related to the self-report nature, including mood-state dependence and social desirability effects (Schneider and Stone, 2016; Van De Mortel, 2008). The psychotic symptoms used to characterise substates were limited to the diary items recorded as part of the Mirorr study. The inclusion of a wider range of psychotic symptoms would provide a more complete characterisation of the symptomatic substates. Perceptual disturbances were only included in post-hoc analyses due to the low endorsement of these items; however, future work should explore their inclusion when applying this methodology to groups further along the psychosis spectrum, where these disturbances may be more prominent. Additionally, future research should explore the inclusion of environmental, social, and treatment factors – either within the HMM or post-hoc analyses – to provide further insight into the factors influencing symptom dynamics. Transition probabilities were assumed constant across the assessment period, however, in practice, they may have varied over time. During the assessment period, the UHR subgroup was allocated to more intensive and specific treatment and the nonclinical subgroup did not undergo treatment. These differing trajectories could have influenced transition patterns. However, this limitation is also not unique to the HMM approach, as many ambulatory assessment methods, such as nontime-varying autoregressive models, similarly assume stable parameters across the assessment period. Finally, we assumed that the transition probabilities were dependent only on the current state and, consistent with the frequency of data collection, that individuals stay in the same state for the entire day. Future extensions to the multilevel HMMs framework may allow for these assumptions to be relaxed.

The present study utilised 90-day baseline daily diary data from the Mirorr study. While the present study provided novel insights into symptom dynamics within individuals early on the psychosis continuum, further work is needed to validate these findings and offer greater insight into their clinical implications and relationship with long-term outcomes. Future analyses will incorporate the 1-year follow-up daily diary data to examine the intraindividual stability of the present findings and determine how changes in symptom dynamics correspond to changes in psychosis risk. Additionally, the relationship between baseline symptom dynamics and transition to full-threshold psychosis over the subsequent four years will be examined.

## Conclusion

The present study identified four symptomatic substates of increasing psychopathological severity in individuals with attenuated psychotic symptoms. Passivity symptoms associated with disorders of self tended to occur only in the context of symptoms of suspiciousness or paranoia and were associated with more severe psychopathological dynamics. Perceptual disturbances were more likely to occur in conjunction with symptoms of unusual thought content. Individuals further along the psychosis continuum exhibited a reduced ability to transition back into less severe symptomatic substates, suggesting a level of psychological inflexibility. These results offer new insights into the dynamics of symptoms in individuals lower on the psychosis continuum that could inform treatment and allow us to refine staging models for greater clinical utility.

## Supporting information

Scott et al. supplementary material Scott et al. supplementary material

## Footnotes

1 In this article, we use the term “attenuated psychotic symptoms” broadly to encompass both the attenuated psychotic symptoms present in ultra-high risk individuals and the psychotic-like experiences that are reported in other clinical and nonclinical groups.

## Supplementary material

The supplementary material for this article can be found at http://doi.org/10.1017/S003329172500056X.
