# Supplementary material for: Characterising symptomatic substates in individuals on the psychosis continuum: a hidden Markov modelling approach
Source: Psychol Med. 2025 Mar 12;55:e82. doi: 10.1017/S003329172500056X (PMC12068681; doi:10.1017/S003329172500056X)
Supplement: Scott et al. supplementary material [file S003329172500056Xsup001.docx]

**SUPPLEMENTARY MATERIAL**

**Contents**

Table S1. Original Dutch translation of daily diary items.

Supplementary Methods: Hidden Markov Models

Figure S1. a) Example of a hidden Markov chain with three hidden states. b) Illustration depicting a realization of the hidden Markov chain over time.

Supplementary Methods. Hyperprior Distribution for Bayesian Model Estimation

Figure S2. Akaike information criterion scores for six multilevel HMMs fitted to 2-7 states.

Figure S3. Distribution of simulated group level emission means generated using the multilevel HMM group-level fitted parameters.

Figure S4. Distribution of simulated individual emission means for suspiciousness, paranoia and broadcasting items against true individual-level emission means.

Figure S5. Distribution of simulated individual emission means for external control, stressed and down items against true individual-level emission means.

Figure S6. Individual level residuals plotted against date for four exemplar individuals.

Figure S7. Histogram of individual level residuals for four exemplar individuals (left-right subgroups 1-4).

Figure S8. Group level emission distributions characterizing the four-substate (left) and five-substate (right) models uncovered with the multilevel HMM.

Figure S9. Individual-level mean item scores across substates.

Figure S10. State sequences for individuals in the Non-Clinical and Mild-PLE subgroups.

Figure S11. State sequences for individuals in the Moderate-PLE and UHR subgroups.

Table S2. Credible intervals (95%) for subgroup transition probabilities, calculated from the MCMC sampler iterations.

Table S3. Differences in substate transition probabilities between subgroups (based on non-overlapping 95% credible intervals).

Figure S12. Distribution of the number of days spent in each substate across individuals within each subgroup.

**Table S1. Diary items.** Items used with the present analyses (21, 31, 42-45, 47, 53) are given in bold.

| **Item** | **Dutch** | **English Translation** | **Response Range** | **Range Description** |
| --- | --- | --- | --- | --- |
| 1 | Ik voel me nu | Right now, I feel | Very unpleasant – Very pleasant | 0 - 100 |
| 2 | Ik voel me nu | Right now, I feel | Very restless/excited – Very quiet/calm | 0 - 100 |
| 3 | Op mijn beste moment van vandaag voelde ik mij | During my best moment of the day, I felt | Very unpleasant – Very pleasant | 0 - 100 |
| 4 | Op mijn beste moment van vandaag voelde ik mij | During my best moment of the day, I felt | Very restless/excited – Very quiet/calm | 0 - 100 |
| 5 | Wanneer was dit beste moment ongeveer? Ergens in de | Around when was this best moment? | Morning, Afternoon, Evening | 1, 2, 3 |
| 6 | Op mijn slechtste moment van vandaag voelde ik mij | During my worst moment of the day, I felt | Very unpleasant – Very pleasant | 0 - 100 |
| 7 | Op mijn slechtste moment van vandaag voelde ik mij | During my worst moment of the day, I felt | Very restless/excited – Very quiet/calm | 0 - 100 |
| 8 | Wanneer was dit slechtste moment ongeveer? Ergens in de | Around when was this worst moment? | Morning, Afternoon, Evening | 1, 2, 3 |
| 9 | Heb je afgelopen nacht goed geslapen? | Did you sleep well tonight? | Not at all – Very well | 0 - 100 |
| 10 | Hoeveel uur heb je afgelopen nacht ongeveer geslapen? | About how many hours did you sleep tonight? | Hours, minutes | 0 - 24 |
| 11 | Heb je vandaag overdag geslapen? (dutjes) | Did you sleep during the day today (naps)? | No (skip to 13), Yes | 0 - 1 |
| 12 | Hoe lang in totaal? | How long in total? | Hours, minutes | 0 - 12 |
| 13 | Ik voelde me vandaag ontspannen | I felt relaxed today | Not at all – Very much | 0 - 100 Positive deactivation |
| 14 | Ik voelde me vandaag kalm | I felt calm today | Not at all – Very much | 0 - 100 Positive deactivation |
| 15 | Ik voelde me vandaag tevreden | I felt satisfied today | Not at all – Very much | 0 - 100 Positive deactivation |
| 16 | Ik voelde me vandaag energiek | I felt energetic today | Not at all – Very much | 0 - 100 Positive activation |
| 17 | Ik voelde me vandaag enthousiast | I felt enthusiastic today | Not at all – Very much | 0 - 100 Positive activation |
| 18 | Ik voelde me vandaag opgewekt | I felt cheerful today | Not at all – Very much | 0 - 100 Positive activation |
| 19 | Ik voelde me vandaag lusteloos | I felt apathetic today | Not at all – Very much | 0 - 100 Negative deactivation |
| 20 | Ik voelde me vandaag moe | I felt tired today | Not at all – Very much | 0 - 100 Negative deactivation |
| **21** | **Ik voelde me vandaag somber** | **I felt down today** | **Not at all – Very much** | **0 - 100 Negative deactivation** |
| 22 | Ik voelde me vandaag angstig | I felt anxious today | Not at all – Very much | 0 - 100 Negative activation |
| 23 | Ik voelde me vandaag onrustig | I felt restless today | Not at all – Very much | 0 - 100 Negative activation |
| 24 | Ik voelde me vandaag prikkelbaar | I felt irritable today | Not at all – Very much | 0 - 100 Negative activation |
| 25 | Ik voelde me vandaag geïrriteerd | I felt irritated today | Not at all – Very much | 0 - 100 Irritation |
| 26 | Ik voelde me vandaag spraakzaam | I felt talkative today | Not at all – Very much | 0 - 100 Spontaneity |
| 27 | Ik voelde me vandaag zelfverzekerd | I felt confident today | Not at all – Very much | 0 - 100 Self-confidence |
| 28 | Ik voelde me vandaag leeg/vlak | I felt empty today | Not at all – Very much | 0 - 100 Flat affect/Anhedonia |
| 29 | Ik voelde me vandaag ongerust | I felt worried today | Not at all – Very much | 0 - 100 Worrying |
| 30 | Ik voelde me vandaag erg speciaal | I felt very special today | Not at all – Very much | 0 - 100 Delusions |
| **31** | **Ik voelde me vandaag wantrouwig** | **I felt suspicious today** | **Not at all – Very much** | **0 - 100 Delusions** |
| 32 | Ik had vandaag het gevoel te kort te schieten | Today I had the feeling of falling short | Not at all – Very much | 0 - 100 Worthlessness |
| 33 | Ik kon vandaag aan wat op mijn pad kwam | Today I could handle what came my way | Not at all – Very much | 0 - 100 Resilience |
| 34 | Ik kon me vandaag goed concentreren | I could concentrate well today | Not at all – Very much | 0 - 100 Concentration |
| 35 | Ik vond mijn leven vandaag de moeite waard | I found my life worthwhile today | Not at all – Very much | 0 - 100 Worthlessness |
| 36 | Ik had vandaag last van lichamelijke klachten | I was bothered by physical symptoms today | Not at all – Very much | 0 - 100 Physical discomfort |
| 37 | Ik had vandaag de neiging iets onbeheersts te doen | Today I had the tendency to do something unrestrained/wild | Not at all – Very much | 0 - 100 Disorganized thoughts |
| 38 | Mijn gedachten lieten me vandaag niet los | My thoughts wouldn’t leave me alone today | Not at all – Very much | 0 - 100 Disorganized thoughts |
| 39 | Mijn gedachten waren vandaag versneld | My thoughts were racing today | Not at all – Very much | 0 - 100 Disorganized thoughts |
| 40 | Mijn gedachten waren vandaag moeilijk te uiten | My thoughts were difficult to express today | Not at all – Very much | 0 - 100 Disorganized thoughts |
| 41 | Er is vandaag iets vreemds met mij of om mij heen gebeurd dat ik moeilijk kon verklaren | Today something strange happened to me or around me that was difficult for me to explain | Not at all – Very much | 1 - 7 Strange impressions/Delusions |
| **42** | **Ik hoorde vandaag stemmen die anderen niet hoorden** | **Today I heard voices that others couldn’t hear** | **Not at all – Very much** | **1 - 7 Hallucinations** |
| **43** | **Ik zag vandaag dingen die anderen niet zagen** | **Today I saw things that others couldn’t see** | **Not at all – Very much** | **1 - 7 Hallucinations** |
| **44** | **Ik had vandaag het gevoel dat anderen me niet mochten** | **Today I had the feeling that others did not like me** | **Not at all – Very much** | **0 - 100 Paranoia** |
| **45** | **Ik had vandaag het gevoel dat anderen mijn gedachten konden lezen** | **I felt that others could read my thoughts today** | **Not at all – Very much** | **0 - 100 Delusions** |
| 46 | Ik voelde me vandaag onwerkelijk | I felt unreal today | Not at all – Very much | 0 - 100 Delusions |
| **47** | **Ik had vandaag het gevoel dat anderen controle over me uitoefenden** | **I felt that others could control me today** | **Not at all – Very much** | **0 - 100 Delusions** |
| 48 | Ik kon vandaag plezier ervaren wanneer er leuke dingen gebeurden | I could experience pleasure when nice things happened today | Not at all – Very much | 0 - 100 Flat affect/Anhedonia |
| 49 | Er kwam vandaag weinig uit mijn handen | I did not get many things done today | Not at all – Very much | 0 - 100 Motivation/Drive |
| 50 | Ik had vandaag zin om dingen te ondernemen | I felt like undertaking something today | Not at all – Very much | 0 - 100 Motivation/Drive |
| 51 | Ik deed dingen ‘op de automatische piloot’, zonder mij erg bewust te zijn van wat ik aan het doen was | I did things on automatic without being conscious of what I was doing today | Not at all – Very much | 0 - 100 Mindfulness |
| 52 | Mijn eetlust was vandaag | My appetite today was | Smaller than normal – Larger than normal | 0 - 100 Appetite |
| **53** | **Hoe gestrest was je vandaag?** | **How stressed were you today?** | **Not at all – Very much** | **0 - 100 Stress** |
| 54 | In welke mate zijn er vandaag positieve gebeurtenissen geweest? | To what extent did positive events happen today? | Not at all – Very much | 0 - 100 Positive events |
| 55 | Hoe plezierig was deze gebeurtenis? | How pleasant was this event? | Neutral – Very pleasant | 0 - 100 Positive events |
| 56 | Hoe belangrijk was deze gebeurtenis voor mij? | How important was this positive event to me? | Very unimportant – Very important | 0 - 100 Positive events |
| 57 | Was deze positieve gebeurtenis gepland? | Was this positive event planned? | No (skip to 59), Yes | 0 - 1 |
| 58 | Ik keek er naar uit | I was looking forward to it | Not at all – Very much | 0 - 100 Positive events |
| 59 | In welke mate zijn er vandaag negatieve gebeurtenissen geweest? | To what extent did negative events happen today? | Not at all – Very much | 0 - 100 Negative events |
| 60 | Hoe onplezierig was deze gebeurtenis? | How unpleasant was this event? | Very unpleasant – Neutral | 0 - 100 Negative events |
| 61 | Hoe belangrijk was deze gebeurtenis voor mij? | How important was this negative event to me? | Very unimportant – Very important | 0 - 100 Negative events |
| 62 | Was deze negatieve gebeurtenis gepland? | Was this negative event planned? | No (skip to 59), Yes | 0 - 1 |
| 63 | Ik zag er tegen op | I dreaded it | Not at all – Very much | 0 - 100 Negative events |
| 64 | Welke gebeurtenis was het meest spannend of stressvol? | Which event was most exciting or stressful? | The negative event, The positive event | 1 - 2 Event stressfulness |
| 65 | Hoe stressvol of spannend was deze gebeurtenis? | How stressful or exciting was this event? | Not at all – Very much | 0 - 100 Event stressfulness |
| 66 | Hoe ben je met deze (stressvolle) gebeurtenis omgegaan? Ik ben hiermee omgegaan door: | How did you cope with this event? I dealt with this by... | Multiple responses (see text in original) | Coping |
| 67 | Hoeveel ben ik vandaag alleen geweest? | How much was I alone today? | Not for a moment – The whole day | 1 - 7 Social context |
| 68 | Ik was liever wat meer in gezelschap geweest | I would have preferred more company | Not at all – Very much | 0 - 100 Social context |
| 69 | Ik vond het gezelschap van vandaag overwegend | I found today’s company mostly | Very unpleasant – Very pleasant | 0 - 100 Social context |
| 70 | Voelde je je vandaag gesteund? | Did you feel supported today? | Not at all – Very much | 0 - 100 Social context |
| 71 | Ik had liever meer steun gevoeld | I would have liked to feel more support | Not at all – Very much | 0 - 100 Social context |
| 72 | Heb je vandaag met iemand een gesprek gevoerd? | Have you had a conversation with someone today? | No (skip to 78), Yes | 0 - 1 |
| 73 | Met wie was dit gesprek? | With whom was this conversation? | Family, Partner, Friend, Other | 1 - 7 Social context |
| 74 | Hoe kritisch was deze persoon naar jou toe? | How critical was this person towards you? | Not at all – Very much | 0 - 100 Expressed emotions |
| 75 | Hoe warm was deze persoon naar jou toe? | How warm was this person towards you? | Not at all – Very much | 0 - 100 Expressed emotions |
| 76 | In welke mate bemoeide deze persoon zich teveel met jou? | To what extent did this person interfere too much with you? | Not at all – Very much | 0 - 100 Expressed emotions |
| 77 | Ik voelde me verbonden met deze persoon | I felt connected with this person | Not at all – Very much | 0 - 100 Social context |
| 78 | Ik heb vandaag de volgende middelen gebruikt: | I have used the following substance today | See original for options | Substance use |
| 79 | Ik ben vandaag lichamelijk actief geweest | I have been physically active today | Not at all – Very much | 0 - 100 Physical activity |
| 80 | Heb je vandaag goed kunnen functioneren? | Were you able to function well today? | Not at all – Very much | 0 - 100 Functioning |
| 81 | Ik heb zin in morgen | I look forward to tomorrow | Not at all – Very much | 0 - 100 Interest/Motivation |

**Supplementary Methods. Hidden Markov Models**

When modelling temporal data, discrete-time HMMs are characterized by a set of discrete sequential observations where each observation is considered as the output of one of several latent states. A schematic of the HMM dependence structure is given in Figure S1. For daily observations, it is assumed that individuals stay within only one state per day. After fitting the HMM, a state sequence can be generated for each individual by assigning each of their daily observations to the most likely associated hidden state. As such, HMMs can be considered akin to traditional clustering algorithms where observations are separated into discrete groups, with the distinction that the assignment now relates to time points within individuals and that assignment is influenced by the assignment of previous and future time points, in addition to the current observation.

*Modelling Assumptions*

Several assumptions were made in fitting a multilevel HMM to the daily diary data:

1. Markov property: future states are conditionally independent of past states, given the present state: if a participant is in state $s_{t}$ on day $t$, the probability of the participant being in state $s_{t+1}$ on day $t$ is independent of the previous substates ${\{s}_{0}$, $s_{1}$, …, $s_{t-1}\}$.
2. Daily diary observations are independent between, and dependent within, participants. However, an observation on any given day is assumed to be conditionally independent of all other observations for that participant, given the hidden state on that day.
3. The discrete-time multilevel HMM assumes 90 equally spaced observations, a reasonable assumption given participants completed a diary entry each evening.
4. The transition probabilities are constant across the assessment period.


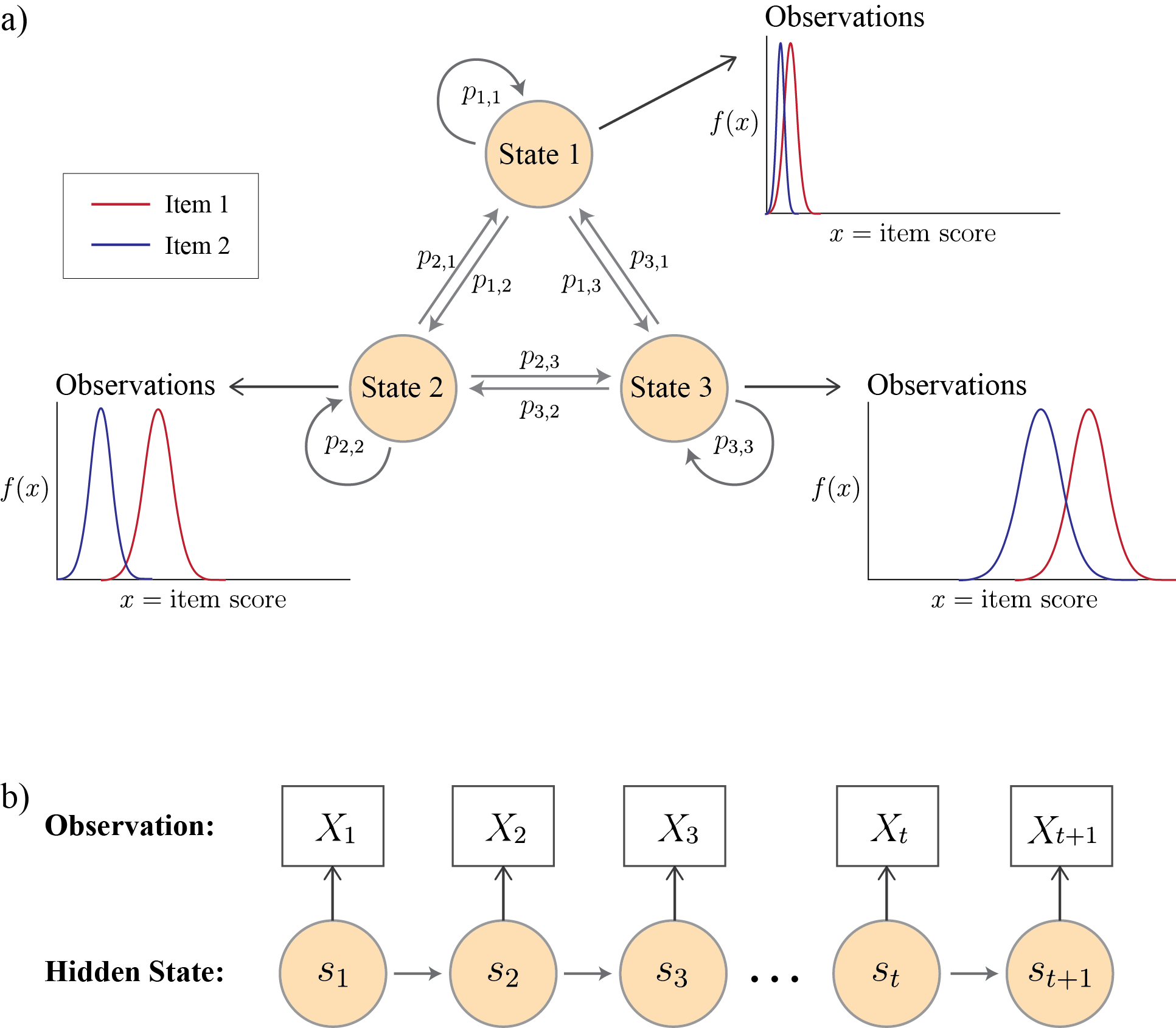


**Figure S1.** **a) Example of a hidden Markov chain with three hidden states**. Arrows between states denote transition probabilities: individuals move from state $i$ on day $t$ to state $j$ on day $t+1$ with probability $p_{i,j}$, for $i, j \boldsymbol{\in\{1, 2, ..., M\}}$ where $\boldsymbol{M}$ is the number of states. The probability of a multivariate observation $x$, corresponding to a set of daily diary item scores, occurring within each state is determined by the probability density function $f(x)$. **b) Illustration depicting a realization of the hidden Markov chain over time.** A sequence of observations is observed, corresponding to a sequence of unobserved hidden states. The observations are dependent over time through their relationship with the hidden states.

**Supplementary Methods. Hyperprior Distribution for Bayesian Model Estimation**

The hyper-prior distribution on the transition probability matrix gamma was left unspecified, as enabled by the mHMMbayes R package. Hyperprior parameters for the continuous emission distribution are required as input (*emiss_hyp_prior*) to the *mHMM* function used to fit the multilevel HMM model and can be specified using the function *prior_emiss_cont*. Further details are provided in the reference manual for the mHMMbayes package (Aarts 2019).

In addition to specifying the number of hidden states (*m*) and dependent variables (*n_dep*), the following hyperparameters were defined for the emission hyperprior:

- emiss_mu0: A list containing n_dep matrices, where each matrix contains the hypothesised hyper-prior means of the Normal emission distribution. Values for emiss_mu0 were specified as the means obtained from an initial single-level HMM fit using the depmixS4 package (Visser & Speekenbrink, 2010).
- emiss_K0 = 1: A list containing n_dep elements denoting the number of hypothetical prior subjects on which the set of hyperprior dependent variable means specified in emiss_mu0 are based. A value of one (for each dependent variable) indicates minimal prior information.
- emiss_V = 100: A list containing n_dep elements corresponding to a vector with length m containing the hypothesised variance between the subject (emission distribution) means, which are assumed to follow an Inverse Gamma hyper-prior distribution. All values were set to 100 to allow for considerable variability across subjects.
- emiss_nu =1: A numeric vector of length one denoting the degrees of freedom of the Inverse Gamma hyper-prior distribution on the between subject variance of the emission distribution means. This value was set to one to reflect substantial uncertainty about the between-subject variance of the emission distribution means.
- emiss_a0 = 1.5, emiss_b0 = 15: Lists containing n_dep elements, where each element is a vector with length m containing the shape (a0) or scale (b0) values of the Inverse Gamma hyper-prior on each of the (fixed over subjects) emission variances of the Normal emission distributions. Repeated values of 1.5 and 15 reflect a weakly informative prior, resulting in an Inverse Gamma distribution with a mean of 30 and an undefined variance, indicating substantial uncertainty. Sensitivity testing of smaller values down to emiss_a0 = 0.001 and emiss_b0 = 0.001 indicated that the modelling results were insensitive to these values. This insensitivity implies that the data is sufficiently informative and that the posterior estimates of the emission variances are primarily driven by the data rather than the specified priors.

Aarts, E. (2019). mHMMbayes: Multilevel hidden Markov models using Bayesian estimation. *R package version 0.1*, *1*.

Visser, I., & Speekenbrink, M. (2010). depmixS4: An R package for hidden Markov models. *Journal of Statistical Software*, 36(7), 1–21.


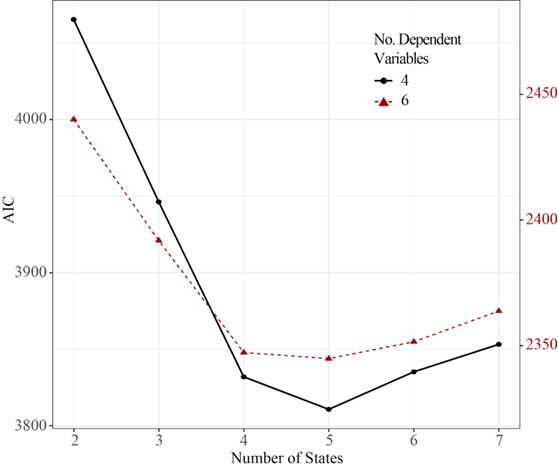


**Figure S2. Akaike information criterion scores for six multilevel HMMs fitted to 2-7 states.** The black line corresponds to scores for the models omitting affective symptoms, whilst scores for the models including affective symptoms are displayed with a red dashed line.

**
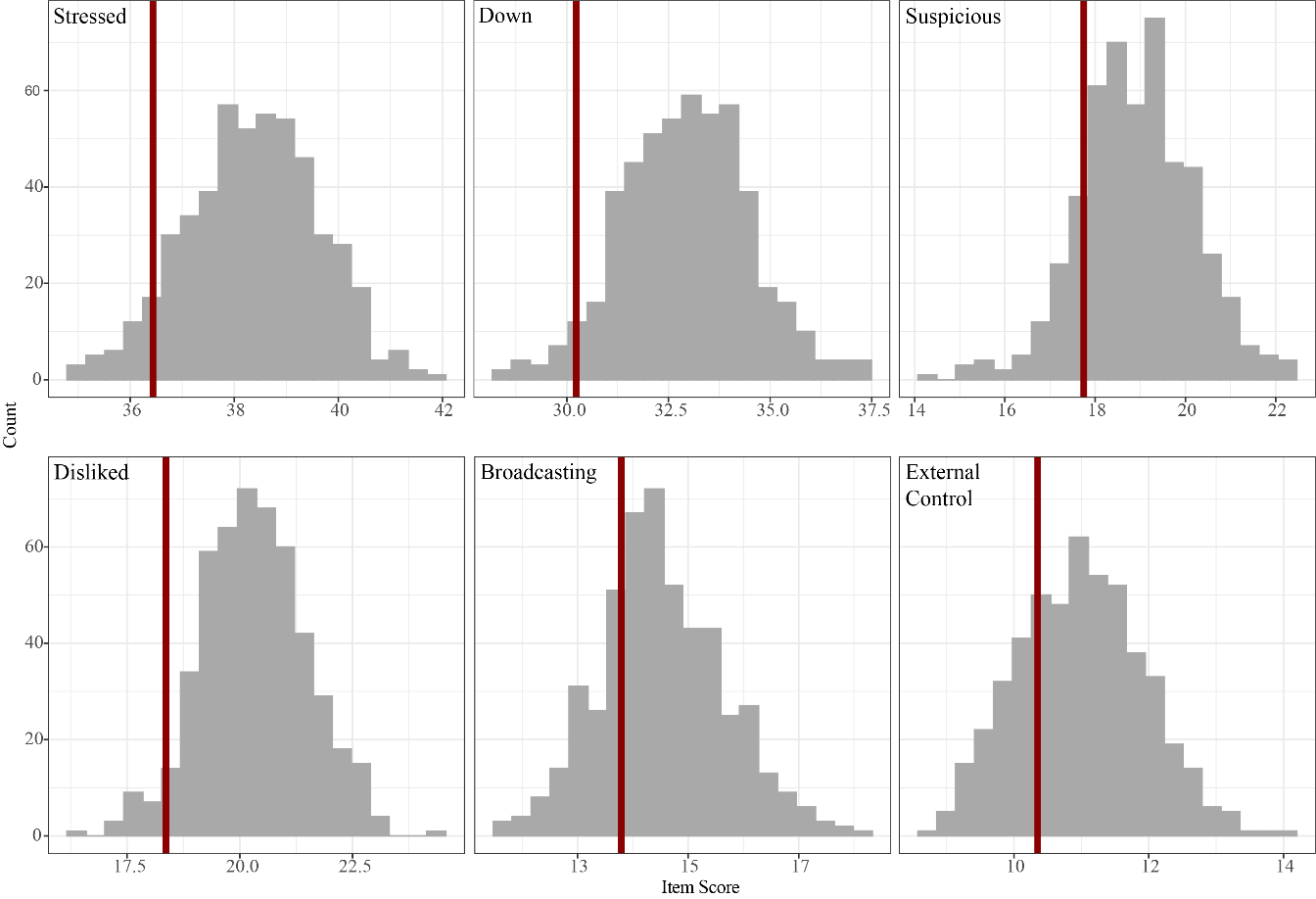
 Figure S3. Distribution of simulated group level emission means generated using the multilevel HMM group-level fitted parameters.** Red line depicts the true empirical aggregate item score means.

**
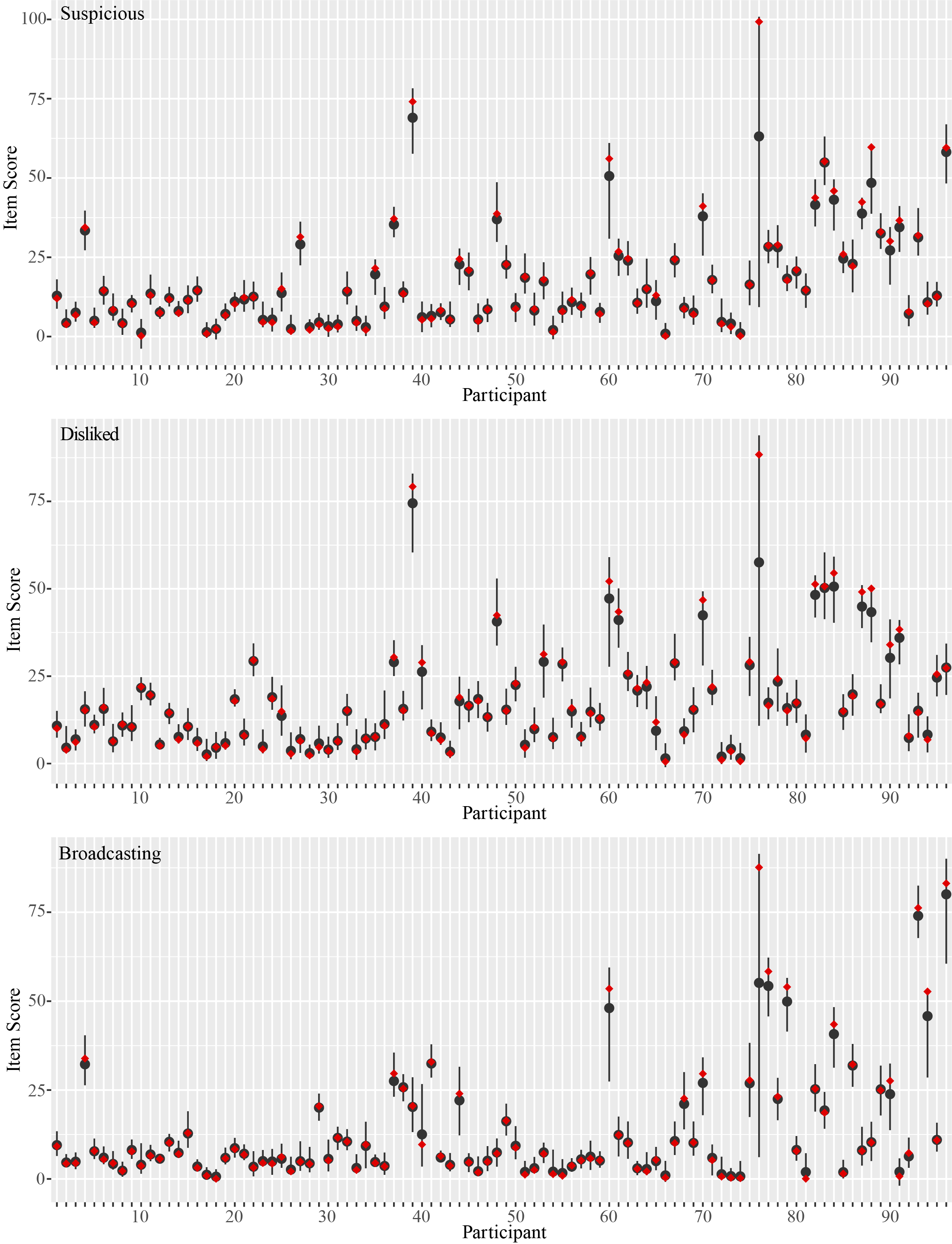
 Figure S4. Distribution of simulated individual emission means for suspiciousness, paranoia and broadcasting items against true individual-level emission means.** Simulated individual means were generated using multilevel HMM fitted parameters.

**
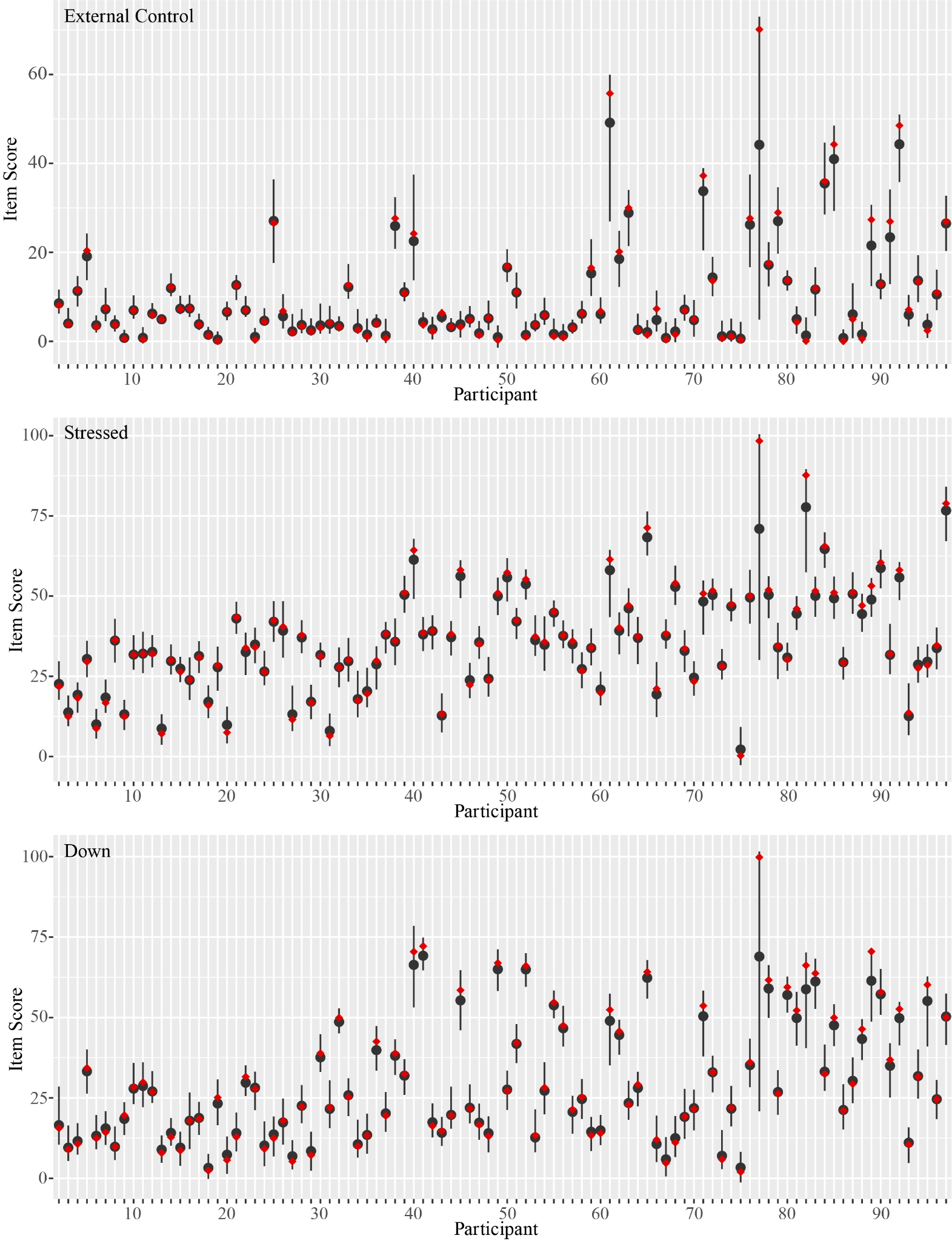
 Figure S5. Distribution of simulated individual emission means for external control, stressed and down items against true individual-level emission means.** Simulated individual means were generated using multilevel HMM fitted parameters.

**
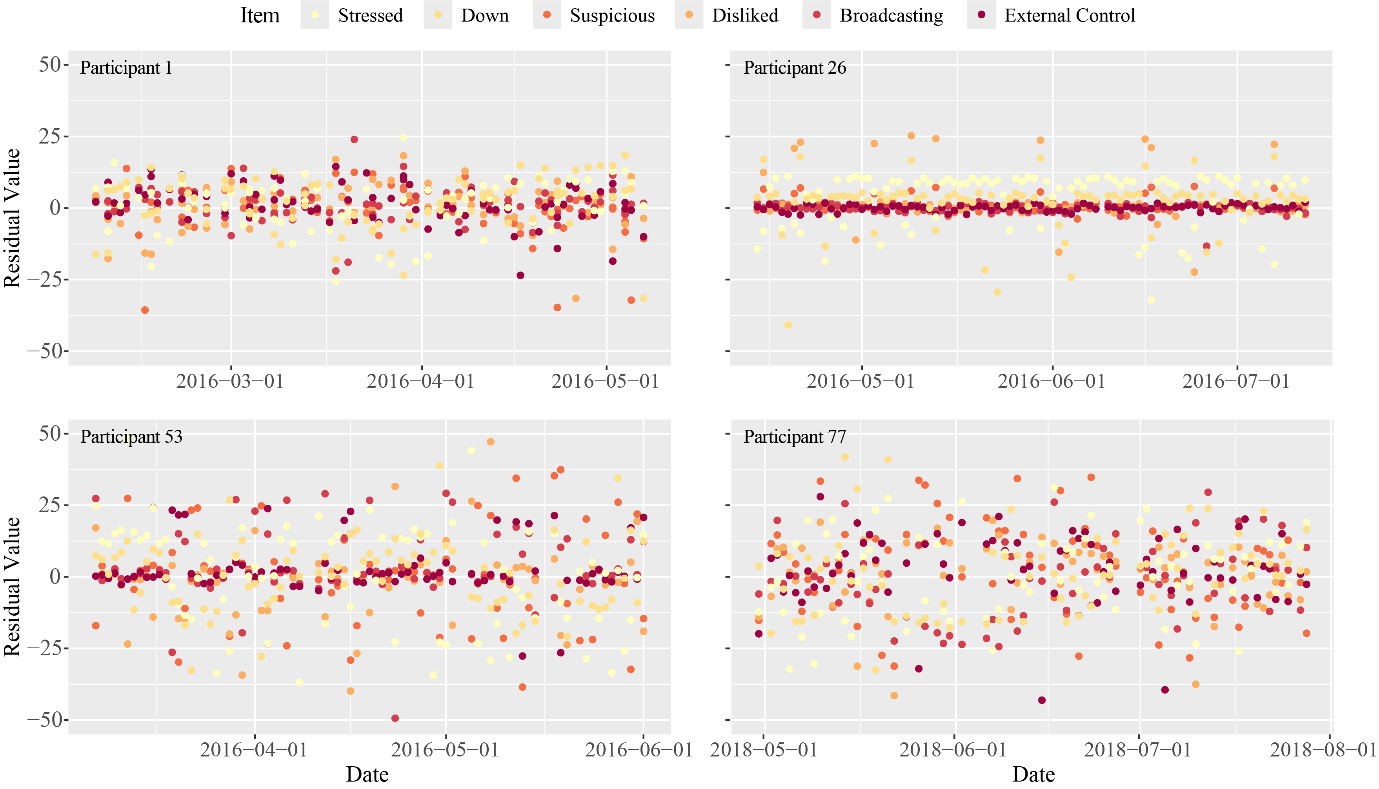
 Figure S6. Individual level residuals plotted against date for four exemplar individuals.** Residuals are zero centred and homoscedastic.


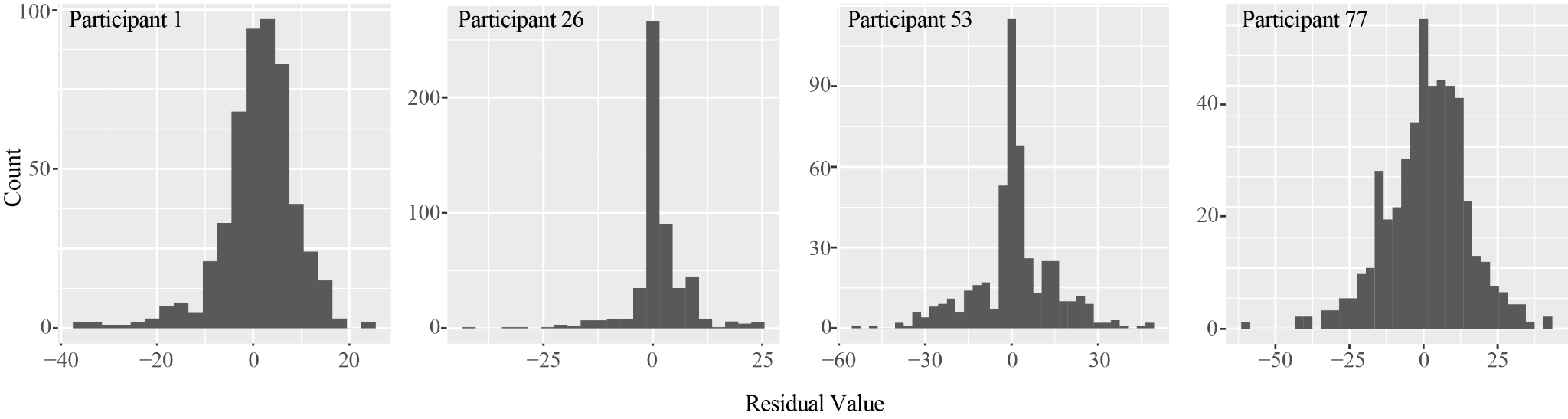


**Figure S7. Histogram of individual level residuals for four exemplar individuals (left-right subgroups 1-4)**. Residuals have been aggregated across the six daily diary items and time.


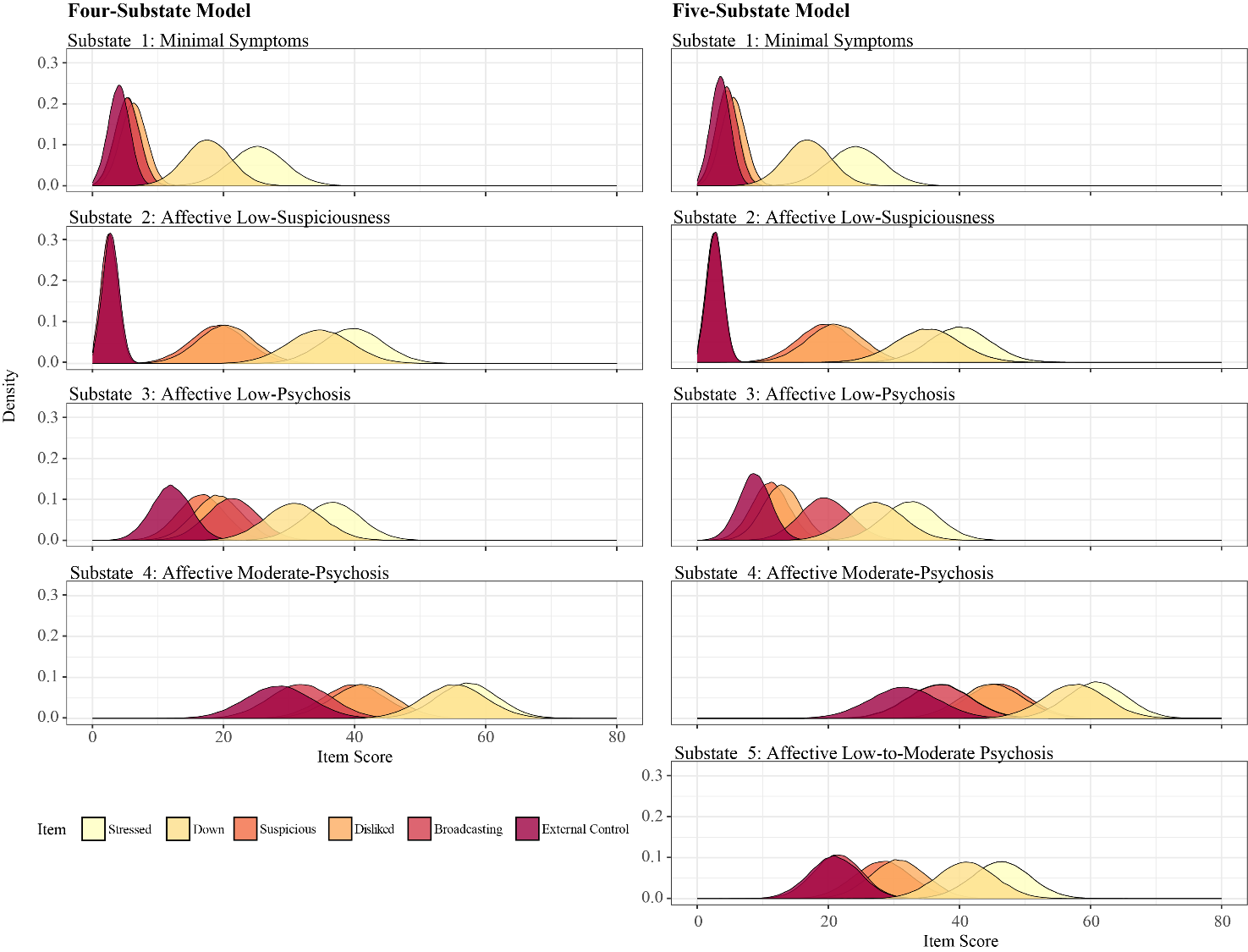


**Figure S8. Group level emission distributions characterizing the four-substate (left) and five-substate (right) models uncovered with the multilevel HMM.**

**
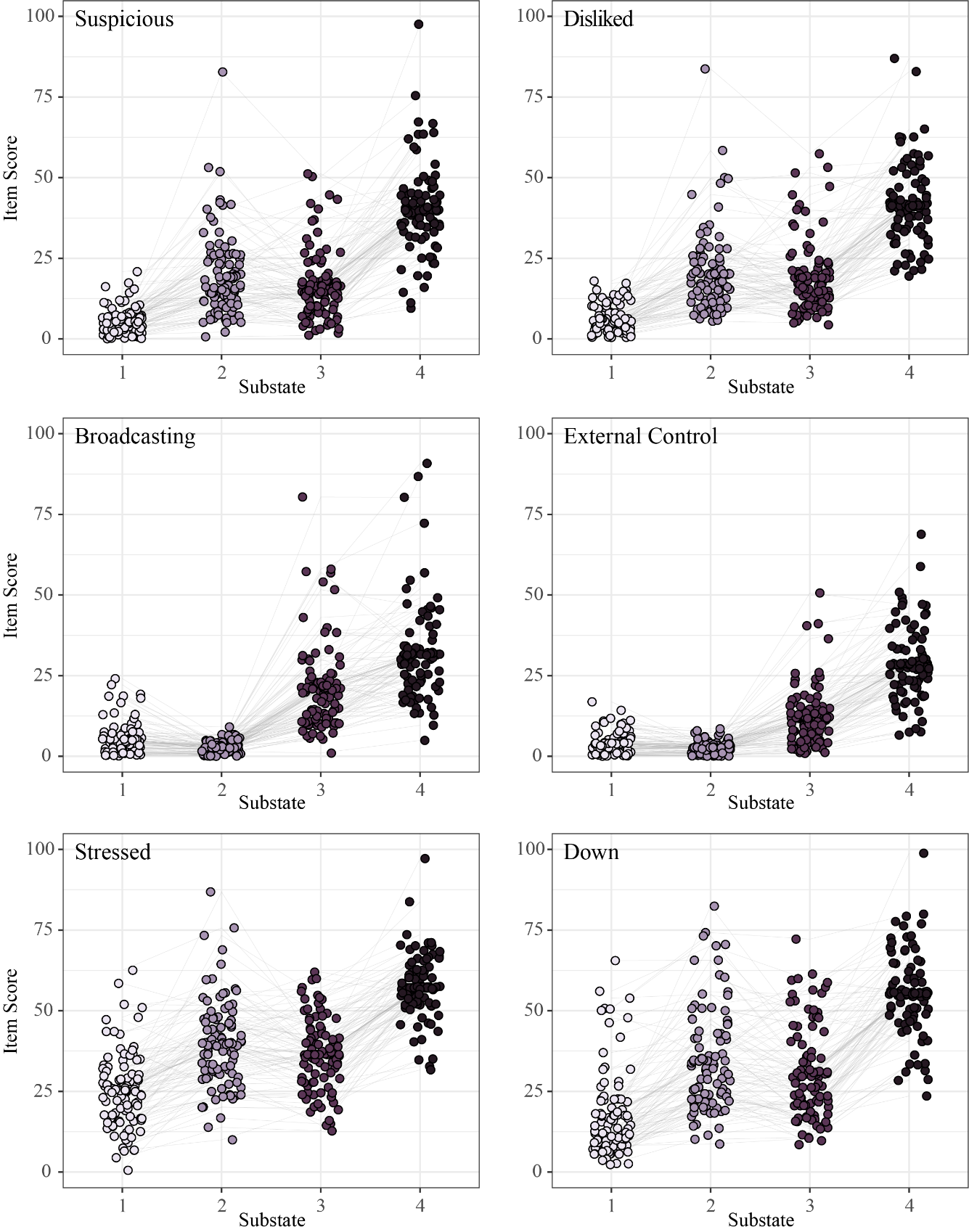
**

**Figure S9. Individual-level mean item scores across substates.** Dots indicate individual emission means for each diary item within each state. Grey lines link the estimated emission means for each individual.

**
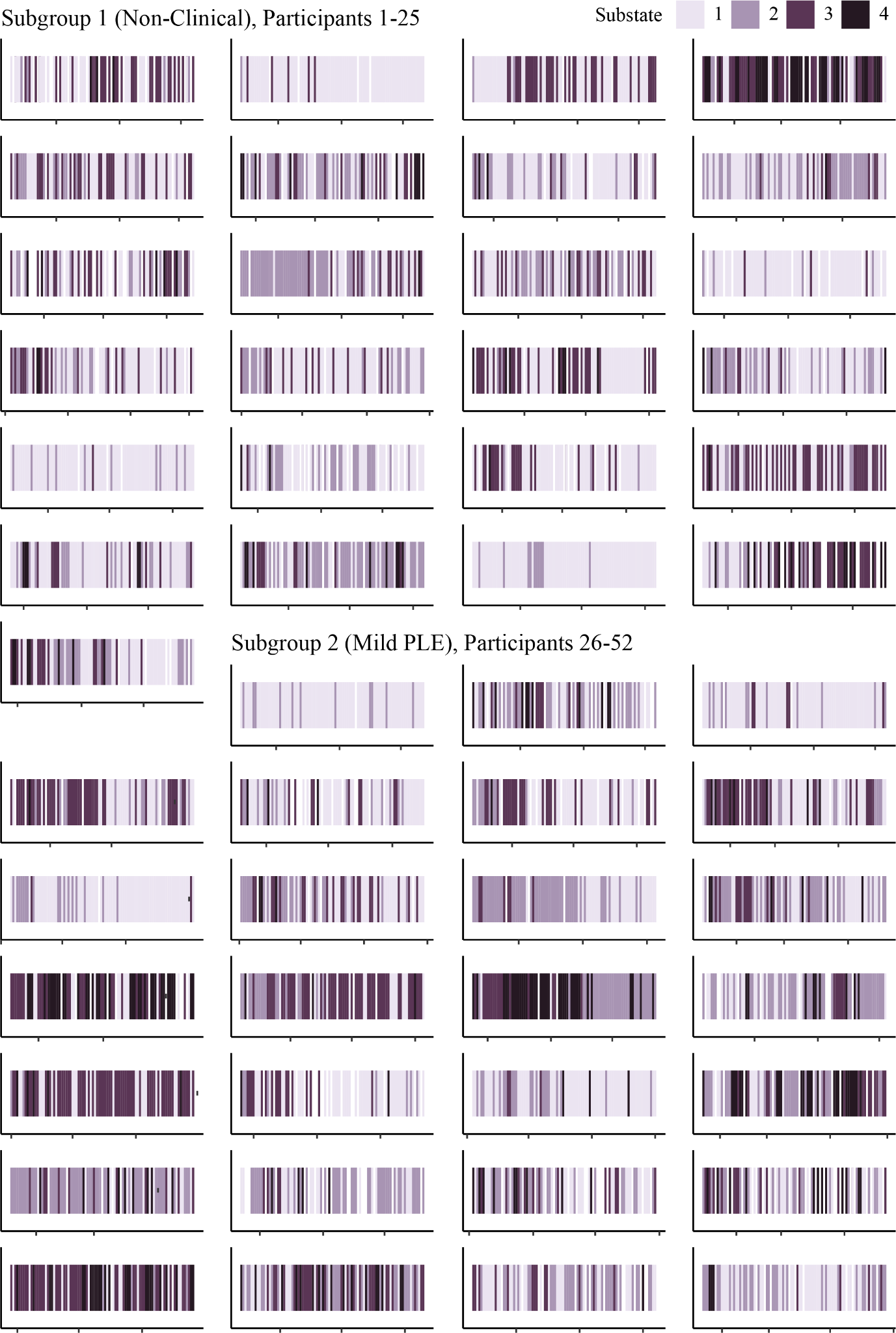
 Figure S10. State sequences for individuals in the Non-Clinical and Mild-PLE subgroups.** State sequences are displayed in order of participant number. Missing state sequence data is displayed in white.

**
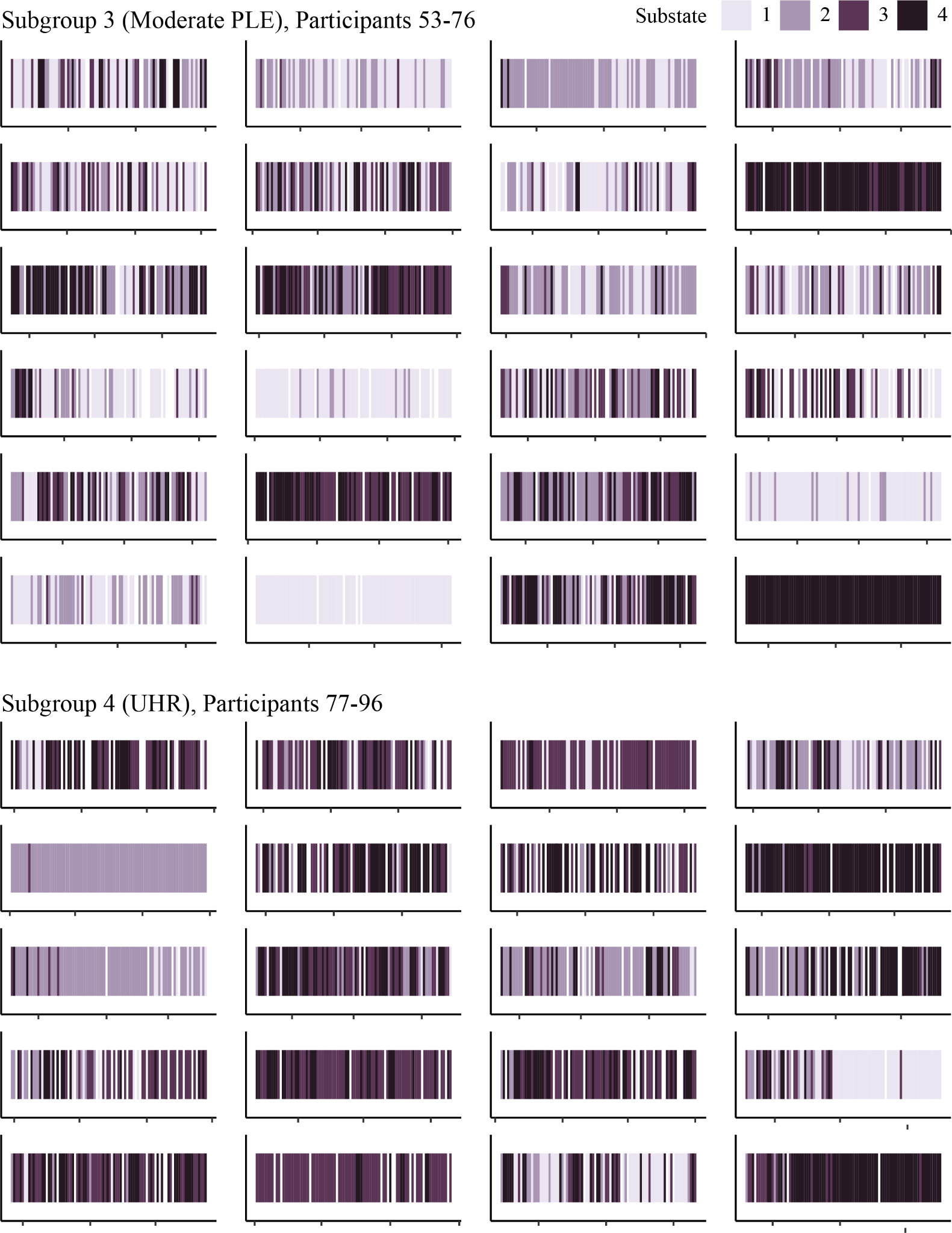
 Figure S11. State sequences for individuals in the Moderate-PLE and UHR subgroups.** State sequences are displayed in order of participant number. Missing state sequence data is displayed in white.

**Table S2. Credible intervals (95%) for subgroup transition probabilities, calculated from the MCMC sampler iterations.**

| Subgroup 1: Non-Clinical | | | | |
| --- | --- | --- | --- | --- |
|  | **To State 1** | **To State 2** | **To State 3** | **To State 4** |
| From State 1 | [0.68, 0.87]* | [0.29, 0.70] | [0.26, 0.67] | [0.01, 0.08] |
| From State 2 | [0.65, 0.89]* | [0.26, 0.56] | [0.07, 0.26] | [0.02, 0.13]* |
| From State 3 | [0.59, 0.88]* | [0.07, 0.33] | [0.20, 0.52] | [0.02, 0.16]* |
| From State 4 | [0.09, 0.33] | [0.07, 0.40] | [0.36, 0.80] | [0.17, 0.56] |
| Subgroup 2: Mild PLE | | | | |
|  | **To State 1** | **To State 2** | **To State 3** | **To State 4** |
| From State 1 | [0.42, 0.76] | [0.15, 0.68] | [0.28, 0.81] | [0.00, 0.15] |
| From State 2 | [0.32, 0.78] | [0.24, 0.61] | [0.06, 0.36] | [0.07, 0.53] |
| From State 3 | [0.22, 0.74] | [0.05, 0.41] | [0.18, 0.85] | [0.06, 0.69] |
| From State 4 | [0.02, 0.23] | [0.02, 0.31] | [0.51, 0.95] | [0.12, 0.78] |
| Subgroup 3: Moderate PLE | | | | |
|  | **To State 1** | **To State 2** | **To State 3** | **To State 4** |
| From State 1 | [0.54, 0.86] | [0.15, 0.78] | [0.09, 0.63] | [0.04, 0.59] |
| From State 2 | [0.24, 0.74] | [0.16, 0.67] | [0.03, 0.21] | [0.13, 0.72]* |
| From State 3 | [0.13, 0.59]* | [0.04, 0.51] | [0.14, 0.65] | [0.16, 0.80]* |
| From State 4 | [0.12, 0.44] | [0.01, 0.25] | [0.42, 0.86] | [0.41, 0.94] |
| Subgroup 4: UHR | | | | |
|  | **To State 1** | **To State 2** | **To State 3** | **To State 4** |
| From State 1 | [0.16, 0.68]* | [0.02, 0.53] | [0.07, 0.82] | [0.06, 0.86] |
| From State 2 | [0.12, 0.53]* | [0.06, 0.56] | [0.03, 0.36] | [0.25, 0.83]* |
| From State 3 | [0.07, 0.40]* | [0.01, 0.34] | [0.16, 0.82] | [0.39, 0.90]* |
| From State 4 | [0.03, 0.28] | [0.01, 0.21] | [0.57, 0.96] | [0.25, 0.91] |

*Denotes a non-overlapping credible interval with another subgroup (pairwise difference between subgroups).

Off-diagonal elements have been normalized to sum to one, meaning that the off-diagonal probabilities represent the likelihood of transitioning into a substate, given that the individual is moving out of their current substate.

**Table S3. Differences in substate transition probabilities between subgroups (based on non-overlapping 95% credible intervals).**

|  |  | **To Substate** | | | |
| --- | --- | --- | --- | --- | --- |
|  |  | **1** | **2** | **3** | **4** |
| **From Substate** | **1** | **Non-Clinical > UHR** | **—** | **—** | **—** |
|  | **2** | **Non-Clinical > UHR** | **—** | **—** | **UHR, Mod-PLE > Non-Clinical** |
|  | **3** | **Non-Clinical > Mod-PLE, UHR** | **—** | **—** | **UHR, Mod-PLE > Non-Clinical** |
|  | **4** | **—** | **—** | **—** | **—** |

**
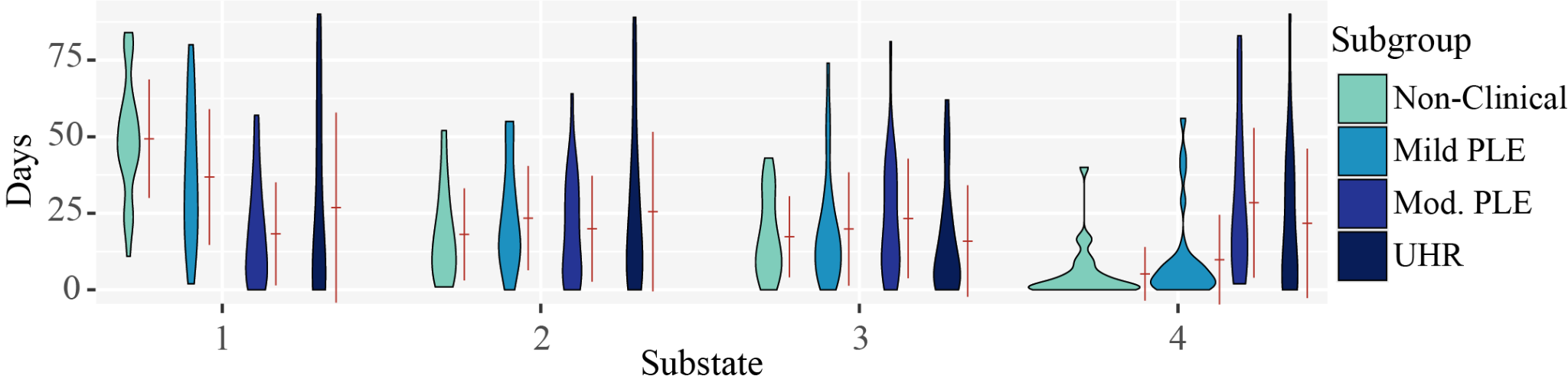
 Figure S12. Distribution of the number of days spent in each substate across individuals within each subgroup.** The mean and standard deviation are shown in red.
